# Synergistic photothermal and antioxidant effects of chiral gold helical nanorods @CeO_2_ nanomaterials for accelerated infected wound healing

**DOI:** 10.1016/j.mtbio.2026.103754

**Published:** 2026-10-15

**Authors:** Jinglei Xu, Qiusha Li, Wen Kan, Wuyi Liu, Chen Liu, Qiuyue Liu, Jie Gao, Kaichuan Yan, Chengzhi Huang, Jian Wang

**Affiliations:** a Key Laboratory of Luminescence Analysis and Molecular Sensing (Southwest University), Ministry of Education, Key Laboratory of Biomedical Analytics (Southwest University), Chongqing Science and Technology Bureau, College of Pharmaceutical Sciences, Southwest University, Chongqing, 400715, PR China; b Key Laboratory of New Drug Screening from Traditional Chinese Medicine, College of Pharmaceutical Sciences, Southwest University, Chongqing, 400715, PR China; c Department of Biological and Chemical Engineering, Chongqing University of Education, Chongqing, 400067, PR China

**Keywords:** Chiral gold helical nanorods, CeO_2_, Antibacterial, ROS scavenging, Triple-synergistic effect

## Abstract

Bacterial infection is a major cause that impedes wound healing. Conventional antibiotic-based treatments, while aimed at suppressing infections, often lead to the emergence of drug resistance. Furthermore, monotherapeutic strategies are frequently inadequate in managing the accompanying inflammation and oxidative stress that follow infection. This work developed chiral gold helical nanorods (AuHNRs)@CeO_2_ nanomaterials with photothermal bactericidal and reactive oxygen species (ROS) scavenging capabilities. D-AuHNRs@CeO_2_ exhibited chiral-selective bioactivity, which preferentially entered damaged L929 mouse fibroblasts cells under oxidative stress, scavenged intracellular ROS, and enhanced wound healing by upregulating Catalase (CAT), transforming growth factor-beta 1 (TGF-β1) in L929 cells, and matrix metalloproteinase-1 (MMP1) in human umbilical vein endothelial cells (HUVECs). Additionally, it effectively shifted RAW264.7 mouse macrophages from the pro-inflammatory M1 phenotype to the pro-repair M2 phenotype, fostering an anti-inflammatory, pro-repair immune microenvironment. Compared with L- and DL-counterparts, D-AuHNRs@CeO_2_ showed superior antibacterial and intracellular antioxidant performance. In a mouse model of infected wounds, D-AuHNRs@CeO_2_ markedly accelerated wound healing and reduced local bacterial burden, highlighting its multifunctional synergistic therapeutic potential. This work presents a novel nanoplatform for the effective management of infected wounds.

**Statement of significance:**

Wound healing is often hindered by a vicious cycle of bacterial infection, excessive inflammation, and oxidative stress. Here, chiral gold helical nanorods decorated with regenerative CeO_2_ (AuHNRs@CeO_2_) was developed. This platform leverages stereoselective interactions to enhance bacterial targeting, converts near-infrared light into heat for photothermal sterilization, and scavenges pathological reactive oxygen species to relieve oxidative stress and inflammation. By integrating multiple therapeutic modalities into one system, this work directly tackles the complex challenge of bacterial wound infection and the subsequent oxidative stress.

## Graphical abstract

**Figure:**
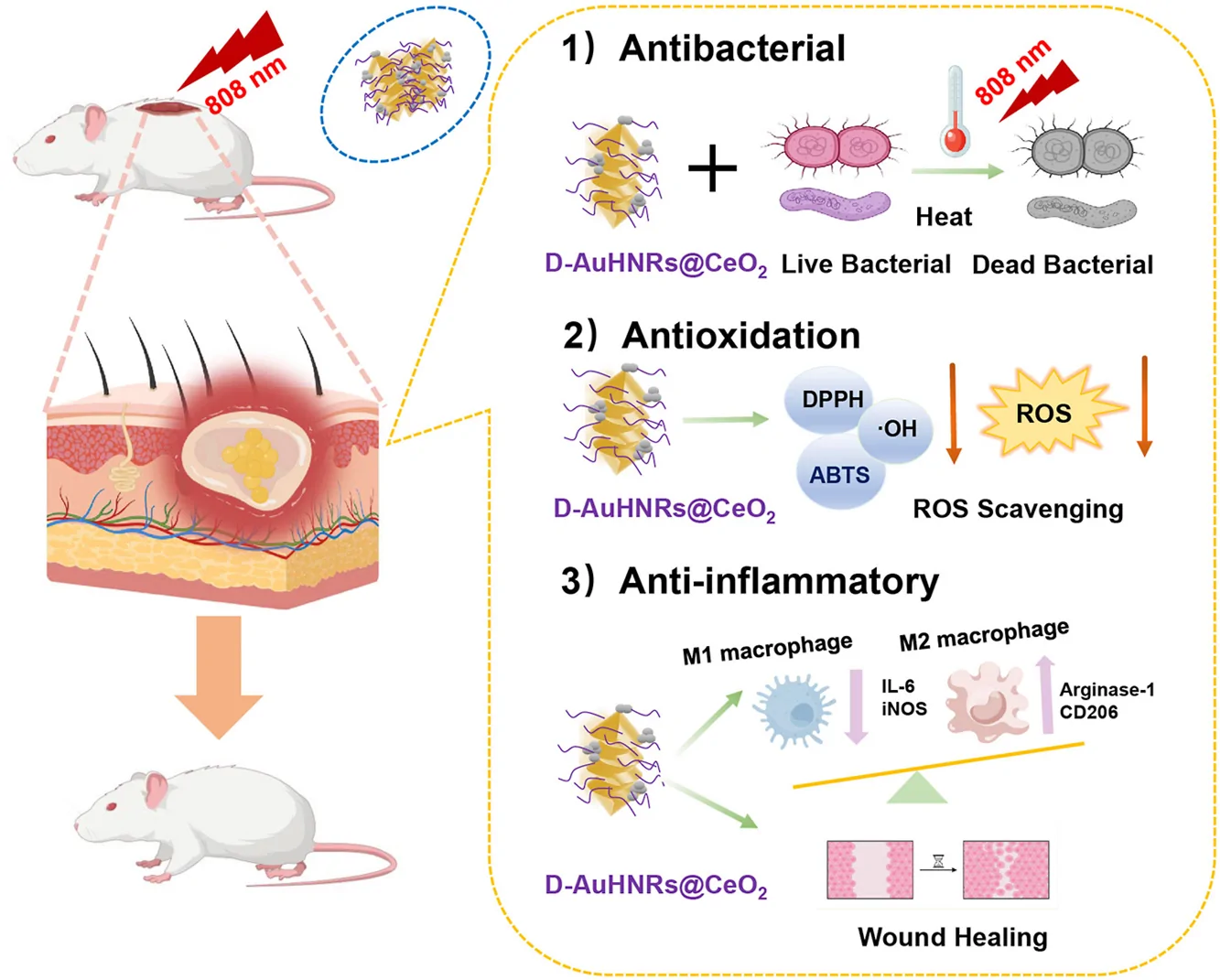

## 1 Introduction

Wound healing is a complex process often hindered by bacterial infection and excessive inflammation [1], [2], [3]. These two factors stimulate the production of free radicals and inflammatory cytokines, disrupt the wound microenvironment, and impede the critical transition from the inflammatory phase to the proliferative phase, leading to delayed healing or even worsening of the wound [4], [5]. The widespread use of conventional antibiotics has accelerated the emergence of multidrug-resistant bacteria, posing significant challenges to clinical treatment, increasing healthcare costs, and elevating patient mortality rates [6]. Compared with conventional antibiotics, nanomaterials allow for rational design strategies, such as size control, surface modification, crystal phase adjustment, and stimuli-responsive functionalization, to achieve targeted interactions with bacterial cells. Moreover, they exhibit a lower tendency to induce resistance and are poised to become a main approach in the treatment of bacterial infections [7], [8].

Chiral nanomaterials exhibit unique property and significant application potential in various fields [9], [10], [11], [12], which participate in stereoselective interactions with chiral biological targets, such as proteins, polysaccharides, and DNA [13], [14], [15]. In antibacterial applications, chiral nanomaterials have emerged as a highly promising platform for developing novel antimicrobial agents due to their unique surface properties and excellent biocompatibility [16]. Since these nanomaterials can be synthesized from chiral precursors like amino acids and polysaccharides, the chiral functional groups on their surfaces enable specific interactions with microbial cell walls or membrane structures [17], [18], [19]. This targeting capability allows chiral nanomaterials to exhibit highly selective antimicrobial effects against specific bacterial or fungal strains, thereby reducing reliance on conventional antibiotics and mitigating the risk of drug resistance development [20], [21], [22].

Furthermore, chiral nanomaterials with photothermal properties can significantly enhance antibacterial activity under light irradiation through localized heat generation or reactive oxygen species (ROS) production [23]. For instance, Chen's group and Hang's group demonstrated that chiral gold nanobipyramids and gold nanostars enable effective near-infrared photothermal therapy (PTT) against bacteria [24], [25]. PTT offers advantages such as minimal side effects and high controllability, and holds promise for overcoming bacterial drug resistance [26]. However, an inherent limitation of PTT is the challenge of managing the oxidative stress generated during treatment and alleviating the subsequent inflammatory response. In addition, photodynamic therapy (PDT) is widely applied in bacterial infection treatment, which works by generating highly cytotoxic ROS to effectively eliminate bacteria. However, as a double-edged sword, ROS can trigger apoptosis or necrosis in pathogenic microorganisms, yet it also intensifies inflammatory responses in surrounding healthy tissues. This duality creates major challenges for its antibacterial applications. In bacterial infection models, elevated ROS levels may aggravate local inflammation and delay tissue repair [27].

At sites of bacterial infection, elevated levels of ROS, including hydrogen peroxide (H_2_O_2_), hydroxyl radicals (·OH), and superoxide anions (·O_2_^−^), induce oxidative stress, which disrupts the wound healing process [28], [29]. Cerium oxide (CeO_2_) nanomaterials have attracted attention due to their excellent antioxidant properties, which are attributed to their reversible Ce^3+^/Ce^4+^ redox transition that enables direct scavenging of multiple ROS [30], [31], [32]. Originally, cerium nitrate was applied for antisepsis of burn wounds. With the development of nanotechnology, research gradually turned to CeO_2_ nanoparticles, which realize ROS regulation at the catalytic level [33]. However, relying only on the catalytic performance of CeO_2_ cannot fully solve the multiple clinical challenges faced by bacterial infection treatment, and there are prominent deficiencies especially in inflammation regulation and antibiotic management. In addition, the multiple catalytic functions of CeO_2_ may produce mutual interference, so precise regulation is needed to construct efficient antibacterial systems [34].

Based on the aforementioned therapeutic requirements, this work developed an integrated nanotherapeutic system that combined chiral recognition with multifunctional synergy (Scheme 1). In this system, chiral gold helical nanorods (AuHNRs) served as the pivotal functional component, exhibiting excellent NIR photothermal conversion performance. Their bactericidal mechanism relied on a purely physical photothermal effect. By absorbing near infrared light and efficiently converting it into localized hyperthermia, they achieved thermal ablation of bacteria without generating any ROS. This approach fundamentally circumvented the inevitable oxidative damage to surrounding healthy tissues caused by conventional photodynamic therapy, which depends on ROS for bactericidal activity. Meanwhile, the microenvironment of infected wounds was usually characterized by abundant endogenous pathological ROS, which could exacerbate local inflammatory responses and disrupt tissue repair processes. The CeO_2_ incorporated into the system was designed to scavenge such harmful ROS via its unique redox properties, thereby exerting anti-oxidant and anti-inflammatory effects and constructing a stable microenvironment conducive to wound healing. In brief, by the synergistic integration of photothermal, antioxidant, and anti-inflammatory actions, the chiral AuHNRs@CeO_2_ nanoplatform demonstrated significant efficacy in promoting wound healing in mouse models of wound infection.

**Scheme 1:**
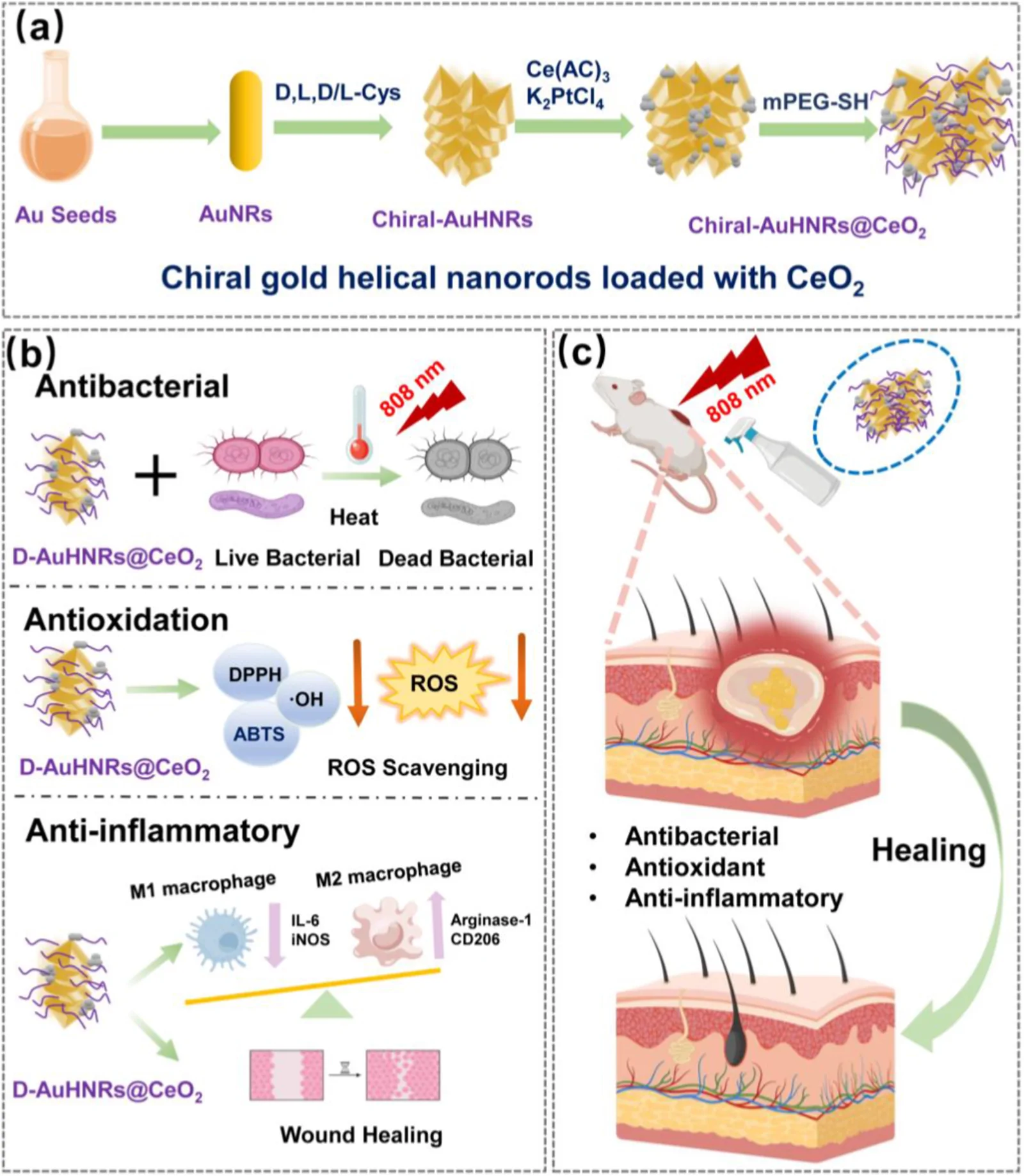
**Schematic illustration of the preparation of chiral AuHNRs@CeO_2_ and its antibacterial application**. (a) Preparation of chiral AuHNRs@CeO_2_. (b) Schematic of the antibacterial, antioxidant and anti-inflammatory effects of chiral AuHNRs@CeO_2_ under 808 nm NIR irradiation. (c) Synergistic therapeutic process of chiral AuHNRs@CeO_2_ in promoting the healing of bacterially infected wounds in a mouse model.

## 2 Experimental methods

### 2.1 *In vitro* photothermal antibacterial activity of chiral AuHNRs and AuHNRs@CeO_2_

The plate count method and live/dead staining experiment were used to evaluate the *in vitro* photothermal antibacterial activity of chiral AuHNRs and AuHNRs@CeO_2_ against *S. aureus* (CMCC (B) 26003) and *E. coli* (ATCC 25922). Briefly, bacterial suspensions (10^6^ CFU/mL) were mixed with chiral AuHNRs or AuHNRs@CeO_2_ at a final concentration of 100 μg/mL. Phosphate-buffered saline (PBS) was used as a negative control in place of the nanomaterials. The mixtures were then divided into two sets for each material: one set was kept in the dark (wrapped in foil and incubated in a shaker at 37 °C, 180 rpm), and the other set was irradiated with an 808 nm laser (0.8 W/cm^2^) for 10 min. After treatment, the mixtures were serially diluted, plated on agar, and incubated overnight. The number of colony-forming units (CFU) was counted. The antibacterial rate was calculated using the following formula: Antibacterial rate (%) = [(CFU__control_ − CFU*_*_sample_)/CFU*_*_control_] × 100%.

### 2.2 Live/dead bacterial staining

After distinct treatments, the bacterial suspension (10^7^ CFU/mL, 500 μL) was co-stained in the dark for 15 min with 500 μL of propidium iodide (PI, 0.03 mM) and N, N-dimethylaniline N-oxide (DMAO, 0.03 mM). After washing three times with PBS to remove unbound probes, the bacteria were observed using fluorescence microscope. All bacteria were effectively stained by DMAO with blue fluorescence, while only dead bacteria with compromised membrane integrity exhibited red fluorescence from PI.

### 2.3 Morphological characterization of bacteria

The morphologies of *E. coli* and *S.aureus* with various treatments were characterized by scanning electron microscopy (SEM). Briefly, 500 μL of bacterial suspension (10^8^ CFU/mL) was inoculated into each well with various treatments and incubated at 37 °C for 12 h under continuous shaking. Afterward, the bacteria were collected and fixed with glutaraldehyde (2.5% (*v/v*), 0.5 mL) at 4 °C for 20 min, and the fixed samples were sequentially dehydrated with gradient ethanol solutions (30-100%, *v/v*) for each 15 min. Finally, the SEM images of samples were captured after sputter coating with gold.

### 2.4 Crystal violet and live/dead staining for biofilms

Crystal violet staining for biofilm assessment was conducted as follows [35]: After incubating bacteria with different treatments for 24 h, the biofilms of *E. coli* and *S. aureus* were fixed with 70% methanol for 20 min. The fixed biofilms were stained with 0.1 mL of 1% (*w/v*) crystal violet solution for 5 min and then washed three times with PBS. After drying, the bound dye was dissolved in 0.5 mL of 33% (*v/v*) acetic acid, and the absorbance at 570 nm was measured using a microplate reader.

$$\text{Biomass}\left(\%\right)=\frac{A_{\text{Control}}‐A_{\text{Sample}}}{A_{\text{Control}}}\times 100\%$$

### 2.5 Intracellular reactive oxygen species detection

**Fluorescence microscopy**: L929 cells were seeded into a 12-well plate and cultured for 24 h until fully adhered. The following control groups were established: Control group: complete medium only; H_2_O_2_ control group control: complete medium containing 2 mM H_2_O_2_; Experimental groups: 2 mM H_2_O_2_ + 100 μg/mL D-, L-, or DL-nanomaterials. All groups were treated simultaneously for 2 h. After discarding the supernatant, serum-free medium containing the fluorescent probe 2′,7′-dichlorodihydrofluorescein diacetate (DCFH-DA, 10 μM) was added, followed by incubation at 37 °C in the dark for 30 min. The culture medium was then removed, and the cells were washed twice with pre-cooled PBS. Images were acquired using fluorescence microscopy.

**Flow cytometry**: L929 cells were seeded into a 6-well plate and cultured for 24 h until fully adhered. The following control groups were established: blank group (complete medium only), probe control group (serum-free medium only), H_2_O_2_ control group (complete medium containing 2 mM H_2_O_2_), and experimental groups (2 mM H_2_O_2_ + 100 μg/mL D-, L-, or DL-nanomaterials). All groups were treated simultaneously for 2 h. After discarding the supernatant, all wells except the blank group were treated with serum-free medium containing the fluorescent probe DCFH-DA (10 μM), followed by incubation at 37 °C in the dark for 30 min. The medium was then removed, and cells were washed twice with pre-cooled PBS, which were collected into 2 mL centrifuge tubes for analysis by flow cytometry.

### 2.6 RT-qPCR analysis

RAW264.7 cells were seeded in a 6-well plate at a density of 2 × 10^5^ cells per well and cultured overnight at 37 °C in a 5% CO_2_ incubator. The cells were stimulated with 1 μg/mL lipopolysaccharide (LPS) for 12 h. Subsequently, chiral AuHNRs@CeO_2_ was added and incubated for an additional 12 h.

L929 cells and HUVECs were seeded in a 6-well plate at a density of 1 × 10^5^ cells per well and cultured overnight at 37 °C in a 5% CO_2_ incubator. The cells were stimulated with H_2_O_2_ (L929 cells: 2 mM and HUVECs: 200 μM) for 12 h. Subsequently, chiral AuHNRs@CeO_2_ was added and incubated for an additional 12 h.

After this treatment, cells were washed with PBS, and total RNA was extracted using TRIzol reagent. The extracted RNA was treated with gDNA Eraser and reverse-transcribed into cDNA using the Evo M-MLV Reverse Transcription Premix kit, following the manufacturer's protocol. Quantitative polymerase chain reaction (qPCR) was then performed using the SYBR Green Pro Taq HS Premix qPCR kit on a real-time PCR system.

### 2.7 Cellular phagocytosis assay

L929 cells were seeded into 24-well culture plates and cultured for 24 h to allow cell attachment. The medium was then aspirated. Cells were subsequently treated for 2 h with complete medium containing 2 mM H_2_O_2_ (experimental groups) or with complete medium alone (control groups). After washing with PBS, all wells were incubated with medium containing 100 μg/mL FITC-labeled chiral AuHNRs@CeO_2_ for 24 h. The medium was removed, cells were washed again, and then stained with 4’,6-diamidino-2-phenylindole (DAPI). Finally, images were acquired with a high-content imaging system (ImageXpress Micro Confocal).

### 2.8 H&E staining

Following euthanasia, tissue samples including the dorsal skin (wound site), heart, liver, spleen, lungs, and kidneys were collected and fixed in 4% paraformaldehyde. The tissues were then embedded in paraffin. Subsequently, the paraffin-embedded tissue blocks were sectioned into 4 μm thick slices. These sections were stained with hematoxylin and eosin (H&E, Servicebio, China) to observe pathological changes in different tissues.

### 2.9 Wound healing assay *in vivo*

To evaluate the wound healing efficacy of chiral nanomaterials, this work established a skin wound infection model using female BALB/c mice. A full-thickness circular skin wound with a diameter of 8 mm was created on the dorsal side of each mouse. The wounds were inoculated with 0.1 mL of *S. aureus* suspension (10^7^ CFU/mL) to establish an infected full-thickness excisional wound model (designated as day 0). Mice with *S. aureus*-infected wounds were randomly divided into four groups: PBS group, PBS + NIR group, chiral AuHNRs@CeO_2_ group, and chiral AuHNRs@CeO_2_ + NIR group. Wounds in each group received corresponding treatments on days 1 and 3. Wound healing progress and mouse body weight were monitored on days 0, 3, 6, 9, and 12, and wound areas were quantified using ImageJ software.

## 3 Results and discussion

### 3.1 Preparation and characterization of D-, L-, and DL-AuHNRs@CeO_2_

The as-prepared gold nanorods (AuNRs) were used as seeds (Fig. S1) with L- and D-cysteine (L-/D-Cys) as chiral inducers (Fig. S2) to synthesize chiral AuHNRs via a seed-regrowth approach (Fig. S3) [9]. Subsequently, chiral AuHNRs@CeO_2_ composites were fabricated with a characteristic absorption peak around 808 nm [36]. During the synthesis, the loading concentration of Ce(Ac)_3_ onto AuHNRs was optimized based on its antibacterial efficacy against *Staphylococcus aureus* (*S. aureus*), which was determined to be 0.05 mM (Fig. S4).

UV-Vis absorption spectra indicated a 13 nm blue shift in the characteristic absorption peak of chiral AuHNRs@CeO_2_ compared to chiral AuHNRs alone, confirming the successful deposition of CeO_2_ onto the AuHNR surface (Fig. 1a). Circular dichroism (CD) and anisotropy factor (g-factor) spectroscopy revealed two distinct chirality-specific signals for both D- and L-AuHNRs, whereas the DL-AuHNRs@CeO_2_ showed no detectable signal (Fig. 1b and S5). This chirality-dependent CD profile was retained in the CeO_2_-loaded composites, namely, D- and L-AuHNRs@CeO_2_ maintained their respective CD signals, while DL-AuHNRs@CeO_2_ remained CD-silent, indicating that the chiral configuration was preserved throughout the composite formation process. Additionally, the systematic characterization results revealed that the g-factors of the chiral AuHNRs@CeO_2_ nanocomposites exhibited no statistically significant alteration after methoxypoly(ethylene glycol) thiol (mPEG-SH) modification (Fig. S6 and Fig. 1c). The average lengths and diameters of D-AuHNRs, L-AuHNRs, D-AuHNRs@CeO_2_ and L-AuHNRs@CeO_2_ were characterized via SEM (Fig. 1d–e and Fig. S7), which revealed a slight reduction in the aspect ratio after CeO_2_ coating (Table S1).

**Fig. 1:**
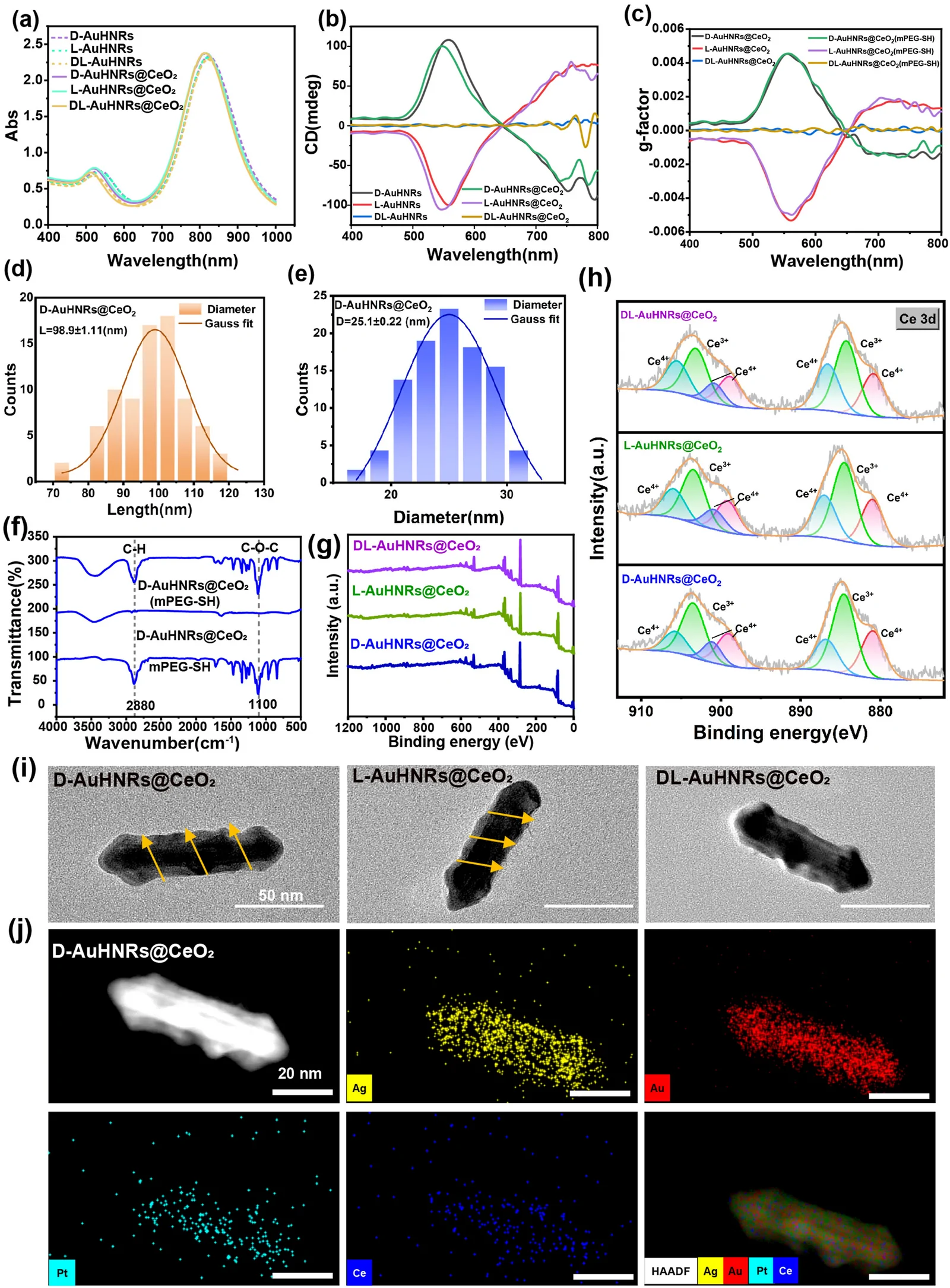
Characterization of chiral AuHNRs@CeO_2_. (a) UV-Vis spectra of chiral AuHNRs and chiral AuHNRs@CeO_2_. (b) CD spectra of chiral AuHNRs and AuHNRs@CeO_2_. (c) g-factor spectra of chiral AuHNRs@CeO_2_ before and after mPEG-SH modification. (d) Length distribution of D-AuHNRs@CeO_2._ (e) Diameter distribution of D-AuHNRs@CeO_2_ (*n* = 100). (f) FTIR spectra of mPEG-SH, D-AuHNRs@CeO2 and D-AuHNRs@CeO2(mPEG-SH). (g) Survey XPS spectra of chiral AuHNRs@CeO_2_. (h) Ce 3d XPS spectra of chiral AuHNRs@CeO_2_. (i) SEM images of chiral AuHNRs@CeO_2_. (j) HRTEM image and elemental mapping of D-AuHNRs@CeO_2_.

Furthermore, FTIR and zeta potential measurements jointly verified the successful PEG modification of the nanoparticles. The PEGylated samples exhibited characteristic PEG absorption bands (C–O–C stretching at ∼1100 cm^−1^ and C–H stretching at ∼2880 cm^−1^) that were absent in bare nanoparticles, directly proving PEG presence on the surface (Fig. S8). Unmodified D-AuHNRs@CeO_2_, L-AuHNRs@CeO_2_ and DL-AuHNRs@CeO_2_ had zeta potentials of +2.86, +3.62 and + 3.97 mV respectively, which changed to −2.55, −1.92 and −1.97 mV after mPEG-SH modification (Fig. S9). This potential reversal, caused by charge shielding from the neutral PEG layer, further confirmed successful PEG grafting on the chiral nanoparticles (Fig. 1f). X-ray photoelectron spectroscopy (XPS) was performed to characterize the chemical states. The high-resolution Ce 3d spectra confirmed the coexistence of Ce^3+^ and Ce^4+^ in chiral AuHNRs@CeO_2_ (Fig. 1g– h and S10) [37], [38], [39]. Moreover, SEM images revealed that all chiral AuHNRs@CeO_2_ composites maintained a similar rod-like morphology (Fig. 1i). Further structural and compositional characterization was conducted using high-resolution transmission electron microscopy (HRTEM) and elemental mapping, which revealed that Au was distributed in well-defined rod-shaped and particulate forms, while the Ce signal was comparatively weak, suggesting a lower abundance or thinner coating of this element (Fig. 1j and S11). These above microscopic and spectroscopic findings provided a comprehensive characterization of the composites, which confirmed both their elemental distribution and chemical states. The colloidal stability of mPEG-SH-functionalized chiral nanomaterials was systematically evaluated in PBS over a 7-day incubation period. UV-Vis absorption spectra, CD spectra, and the corresponding dissymmetry g-factor were recorded at predefined time points of Day 0, Day 1, Day 3, and Day 7. No significant shifts in the characteristic UV-Vis absorption peaks, no measurable attenuation of CD signal intensity, and negligible variation in the g-factor were observed across the entire test duration (Figs. S12–S13). These consistent spectroscopic results confirmed that the mPEG-SH-functionalized chiral AuHNRs@CeO_2_ nanocomposites possessed excellent structural and chiroptical stability in physiologically relevant aqueous media.

### 3.2 Photothermal performance and reactive oxygen species scavenging efficiency of chiral AuHNRs@CeO_2_

The photothermal effects of D-, L-, and DL-AuHNRs, as well as their CeO_2_-loaded counterparts (AuHNRs@CeO_2_), were evaluated by monitoring temperature changes under 808 nm NIR laser irradiation. At a power density of 0.8 W/cm^2^ for 10 min, the photothermal conversion efficiencies were 52.6%, 52.3%, and 48.4% for D-, L-, and DL-AuHNRs, respectively, and 47.6%, 46.9%, and 42.8% for D-, L-, and DL-AuHNRs@CeO_2_ (Fig. 2a – b and S14). Although the incorporation of CeO_2_ partially attenuated the NIR absorption and heat generation of the chiral AuHNRs, the temperature increase remained within the ideal range for PTT applications [35], [40]. Further assessment of the photothermal performance revealed that the temperature rise correlated positively with increasing concentrations of the chiral nanomaterials (Fig. 2c–d and S15). Similarly, the temperature elevation was more pronounced at higher laser power densities (Fig. 2e–f and Fig. S16). Moreover, the chiral nanomaterials exhibited excellent photothermal recycling stability, maintaining effective heat generation over five laser on/off cycles (Fig. 2g–h and S17).

**Fig. 2:**
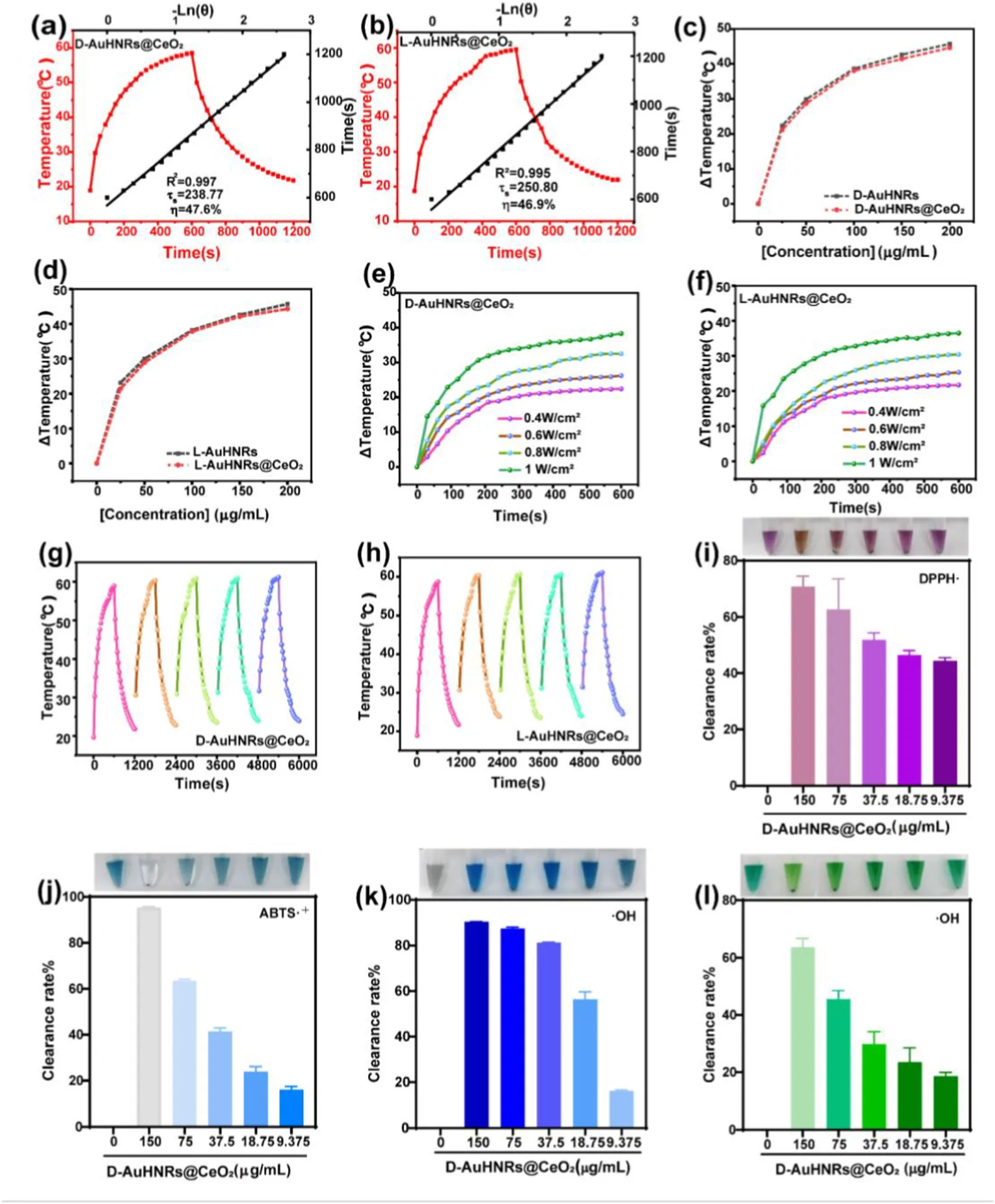
Photothermal performance and ROS scavenging efficiency of chiral AuHNRs@CeO_2_. Temperature variation of (a) D-AuHNRs@CeO_2_ and (b) L-AuHNRs@CeO_2_ solution under cyclic NIR laser irradiation (808 nm, 0.8 W/cm^2^), along with the corresponding -lnθ versus time plot derived from the cooling phase over 10 min. Temperature elevation profiles of (c) D-AuHNRs@CeO_2_ and (d) L-AuHNRs@CeO_2_ at various concentrations under 808 nm irradiation (0.8 W/cm^2^, 10 min). Temperature elevation curves of (e) D-AuHNRs@CeO_2_ and (f) L-AuHNRs@CeO_2_ under different laser power densities (808 nm, 10 min). Evaluation of the thermal stability of (g) D-AuHNRs@CeO_2_ and (h) L-AuHNRs@CeO_2_ over five consecutive laser on-off cycles (0.8 W/cm^2^, 10 min per cycle). (i) DPPH· scavenging rate. (j) ABTS·^+^ scavenging rate. (k) ·OH scavenging rate measured by MB method. (l) ·OH scavenging rate determined via TMB method.

Bacterial infection-induced inflammatory responses stimulate the excessive production of ROS. The explosive accumulation of these free radicals disrupts the redox homeostasis at the infection site, aggravates tissue oxidative damage and inflammatory cascades, and ultimately impedes the healing process of infected wounds [41], [42]. The 1,1-diphenyl-2-picrylhydrazyl radical (DPPH·) and the 2,2′-azinobis (3-ethylbenzothiazoline-6-sulfonic acid) radical (ABTS·^+^) were employed to assess free-radical scavenging activity. With increasing concentrations of chiral AuHNRs and chiral AuHNRs@CeO_2_, the scavenging rate increased accordingly. After loading CeO_2_, D-, L-, and DL-AuHNRs all exhibited improved scavenging rates compared to their unloaded counterparts (Fig. 2i and S18). A similar trend was observed in the ABTS·^+^ assay [43]. The scavenging rate was concentration-dependent. After CeO_2_ loading, the scavenging rate of chiral AuHNRs was enhanced by 18.2% compared to that of single-component chiral AuHNRs (Fig. 2j and S19). The ·OH-scavenging capacity was evaluated using the methylene blue (MB) degradation and the 3,3′,5,5′-tetramethylbenzidine (TMB) oxidation assays based on a Fenton-reaction system. As expected, the ·OH-mediated degradation of MB was markedly suppressed. In contrast, single-component chiral AuHNRs exhibited notable radical-scavenging activity only at high concentrations, indicating that CeO_2_ loading significantly enhanced the ·OH-scavenging efficiency of AuHNRs (Fig. 2k and S20). Similarly, in the TMB assay, the color of the TMB solution gradually faded with increasing material concentration in the presence of chiral AuHNRs. The CeO_2_-loaded chiral AuHNRs showed a 38.9% improvement in ·OH scavenging efficiency compared to the unloaded AuHNRs (Fig. 2l and S21). Finally, the salicylic acid colorimetric method was used for further validating the ·OH-scavenging capability of AuHNRs@CeO_2_, yielding results consistent with the MB and TMB assays (Fig. S22). These results confirmed the potent free radical scavenging ability of the nanomaterials, which was significantly enhanced by CeO_2_ loading.

### 3.3 Antibacterial performance of chiral AuHNRs@CeO_2_

Prior to *in vitro* antibacterial testing, the cytocompatibility of the nanomaterials was first evaluated with a hemolysis assay. The unmodified nanomaterials exhibited a hemolysis rate exceeding 5% (Fig. S23), whereas after modification with mPEG-SH, even under 808 nm NIR irradiation and at a concentration of 100 μg/mL, the hemolysis rate was reduced to below 5%, indicating that this modification significantly improved the hemocompatibility of the materials. These results further confirmed the necessity of mPEG-SH surface modification for enhancing the biosafety of such nanomaterials (Fig. S24) [44].

The *in vitro* antibacterial efficacy of chiral AuHNRs and chiral AuHNRs@CeO_2_ against two representative bacterial strains, Gram-negative *Escherichia coli* (*E. coli*) and Gram-positive *S*. *aureus*, was evaluated with the standard plate counting method. The results showed that in the absence of NIR irradiation and against *S*. *aureus*, D-AuHNRs and D-AuHNRs@CeO_2_ exhibited superior antibacterial efficiency compared to their L- and DL-type counterparts. Against *E. coli*, only the CeO_2_-loaded D- and L-type nanomaterials (i.e., D- and L-AuHNRs@CeO_2_) showed detectable antibacterial activity, which was consistent with expectations. Upon NIR laser irradiation, all experimental groups demonstrated excellent antibacterial efficacy against *E. coli* and *S. aureus* (Fig. 3a–c and S25).

**Fig. 3:**
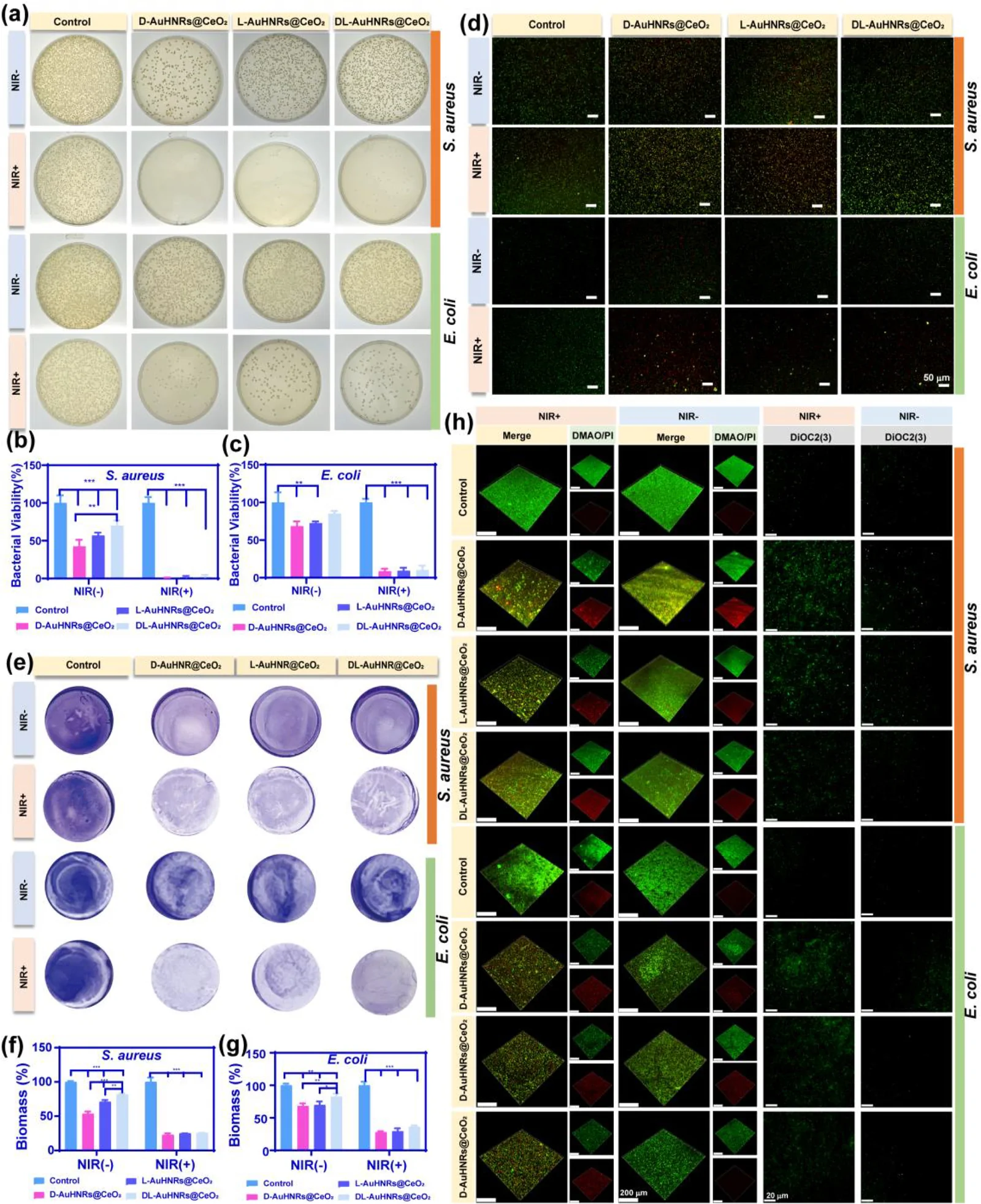
*In vitro* antibacterial activity of chiral AuHNRs@CeO_2_ with or without NIR irradiation. (a) Representative images of agar plates inoculated with *S. aureus* and *E. coli* after the indicated treatments. (b) Survival rate of *S. aureus* after treatment. (c) Survival rate of *E. coli* after treatment. (d) Live/dead staining of *S. aureus* and *E. coli* after treatment (green, live bacteria; red, dead bacteria; scale bar: 50 μm) (e) Crystal violet staining assay of *S. aureus* and *E. coli* after the indicated treatments. (f) Viability assessment of *S. aureus* biofilms after treatment; (g) Viability assessment of *E. coli* biofilms after treatment. (h) 3D confocal microscopy reconstruction images showing structural changes in bacterial biofilms after different treatments (scale bar: 200 μm) and DiOC2 (3) fluorescence staining detecting bacterial membrane potential changes (scale bar: 20 μm; ***p < 0.001, **p < 0.01, *p < 0.05). (For interpretation of the references to color in this figure legend, the reader is referred to the Web version of this article.)

To further validate these findings, bacterial viability was assessed with live/dead staining based on the dual-fluorescence system of N-dimethylaniline N-oxide (DMAO) and propidium iodide (PI). The results confirmed the efficient antibacterial effects of chiral AuHNRs and AuHNRs@CeO_2_ under NIR irradiation (Fig. 3d and S26). After staining and image merging, control groups exhibited green fluorescence, whereas nearly all bacteria in the D-AuHNRs + NIR and D-AuHNRs@CeO_2_ + NIR groups displayed merged red or yellow fluorescence, indicating extensive bacterial death. Under NIR irradiation, the D-AuHNRs group demonstrated superior antibacterial efficacy compared to the L-type and DL-type groups, which was consistent with the plate counting results. In addition, biofilm-infected wounds remain a major clinical challenge, as biofilm infections and persistent inflammation hinder conventional therapies from dynamically adapting to the evolving wound microenvironment [45]. Chiral AuHNRs and AuHNRs@CeO_2_ also demonstrated excellent performance in disrupting bacterial biofilm structures. Crystal violet staining assays revealed substantial biofilm formation in the control groups, while groups treated with chiral AuHNRs + NIR and AuHNRs@CeO_2_ + NIR under 808 nm laser irradiation displayed a remarkable decrease in crystal violet absorbance, indicating a significant reduction in biofilm biomass. These results suggested that NIR laser irradiation enhanced the biofilm-disrupting effect of chiral AuHNRs and AuHNRs@CeO_2_. Moreover, even in the absence of light exposure, the D-type nanomaterial exhibited superior biofilm-disrupting capability compared to the DL-type (Fig. 3e–g and S27). The eradication of mature biofilms (pre-cultured for 48 h) formed by *S. aureus* and *E. coli* was further evaluated. Crystal violet staining showed that the PBS control group exhibited significantly higher biofilm biomass. Under 808 nm NIR irradiation, the D/L/DL-AuHNRs@CeO_2_ + NIR groups showed significantly reduced crystal violet absorbance, indicating a marked decrease in biofilm biomass. Without NIR irradiation, the chiral AuHNRs@CeO_2_ exhibited stronger eradication activity against *S. aureus* biofilms than against *E. coli* biofilms, with the D-type outperforming the L- and DL-type (Fig. S28). 3D confocal microscopy reconstruction visually confirmed biofilm inhibition (Fig. 3h) and disruption (Fig. S29). The control group showed intact, dense biofilms, while NIR-irradiated groups exhibited structural loosening and reduced thickness. D-AuHNRs@CeO_2_ + NIR group showed the most significant disruption, consistent with crystal violet staining. DiOC2(3) staining revealed mild membrane depolarization (partial green fluorescence) without NIR irradiation, whereas the D-AuHNRs@CeO_2_ + NIR group exhibited nearly complete depolarization, indicating markedly enhanced membrane damage (Fig. 3h).

### 3.4 Antibacterial mechanism of chiral AuHNRs@CeO_2_

SEM images of bacterial morphology revealed that both the control group and the control + NIR group maintained intact bacterial structures with smooth surfaces. In contrast, bacteria treated with chiral AuHNRs and AuHNRs@CeO_2_ exhibited slight structural damage. Under NIR irradiation, bacteria treated with chiral AuHNRs and AuHNRs@CeO_2_ suffered severe damage, characterized by collapsed cell membranes and leakage of intracellular contents. Notably, in the chiral AuHNRs@CeO_2_ + NIR and chiral AuHNRs@CeO_2_ groups, distinct chiral AuHNRs@CeO_2_ nanoparticles were observed to attach onto *S. aureus* cells (Fig. 4a). In contrast, no such attachment was detected on *E. coli*, even though structural damage still occurred (Fig. 4b). In addition, no nanoparticle attachment was observed in either *E. coli* or *S. aureus* groups treated with chiral AuHNRs (Fig. S30). This differential behavior may be attributed to the CeO_2_ coating, whose irregular shape and rough edges could cause physical damage to the bacterial membrane, particularly in Gram-positive bacteria, such as *S. aureus* [46], [47].

**Fig. 4:**
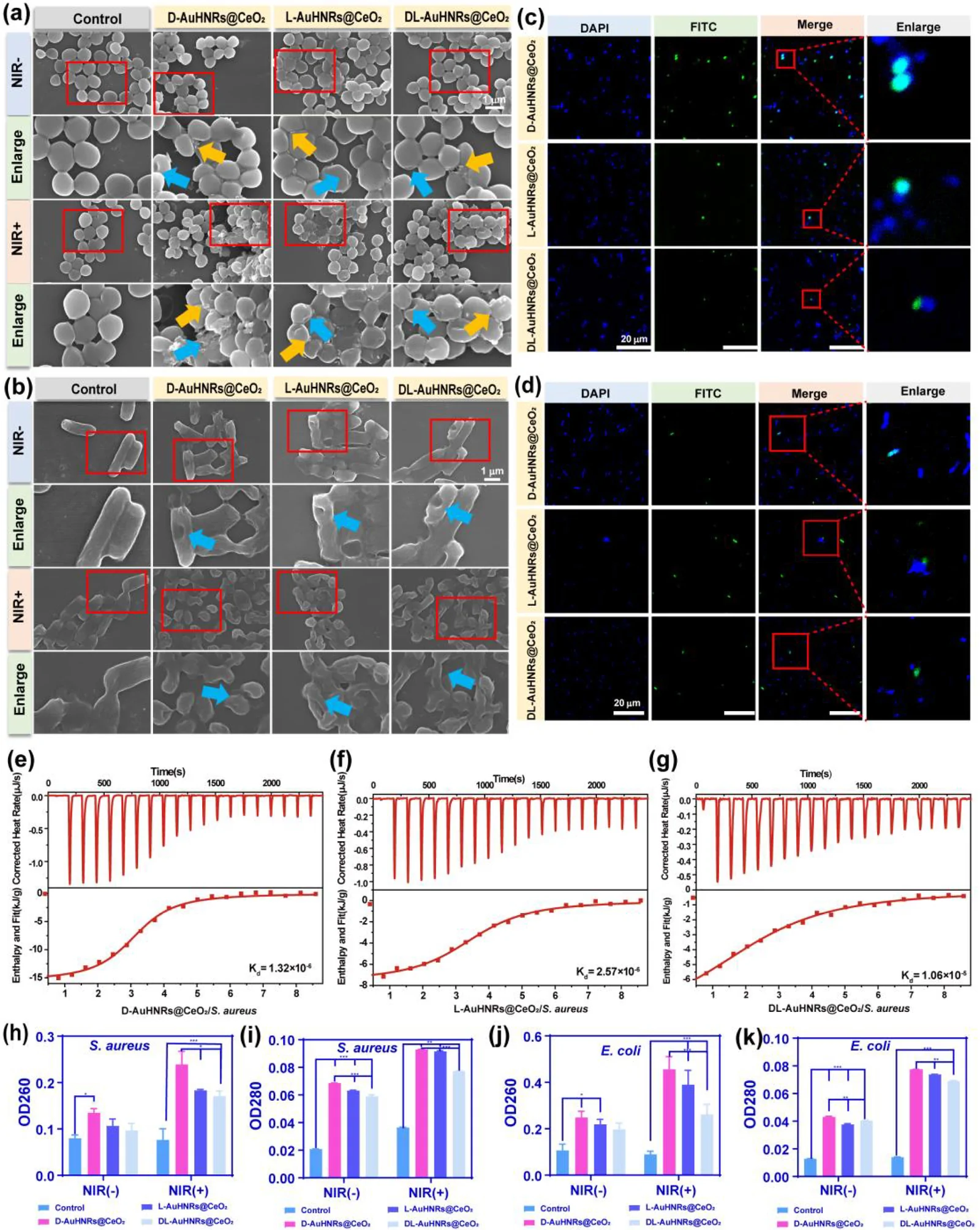
Mechanistic investigation of the antibacterial activity of chiral AuHNRs@CeO_2_ with or without NIR irradiation. (a) SEM images of *S. aureus* after treatment (scale bar: 1 μm; blue arrows, membrane collapse/damage; yellow arrows, bound nanoparticles). (b) SEM images of *E. coli* after treatment (scale bar, 1 μm; blue arrows, membrane collapse/damage). (c) After material treatment, confocal fluorescence imaging was performed on *S. aureus* and chiral AuHNRs@CeO_2_. (d) After material treatment, confocal fluorescence imaging was performed on *E. coli* and chiral AuHNRs@CeO_2_. (Scale bar:20 μm, the red dotted line part is the enlarged area of each bacterial image). ITC titration curves and S-shaped binding isotherms of (e) D-AuHNRs@CeO_2_, (f) L-AuHNRs@CeO_2_, and (g) DL-AuHNRs@CeO_2_ with *S. aureus* at 25 °C. The top panels show the raw thermograms (heat rate vs. time), and the bottom panels show the integrated binding isotherms (normalized heat vs. molar ratio). The solid lines represent the best fit using a one-site binding model. (h) Nucleic acid leakage from *S. aureus* after treatment. (i) Protein leakage from *S. aureus* after treatment. (j) Nucleic acid leakage from *E. coli* after treatment. (k) Protein leakage from *E. coli* after treatment (***p < 0.001, **p < 0.01, *p < 0.05). (For interpretation of the references to color in this figure legend, the reader is referred to the Web version of this article.)

In light of the above observations, further assays were conducted to assess the temperature increase after co-incubation of bacteria with the nanomaterials and subsequent centrifugation to remove unbound chiral nanomaterials. The results demonstrated that D-type nanomaterials produced a more pronounced heating effect. This difference may arise from the tendency of D-AuHNRs@CeO_2_ to localize near the cell wall of *S. aureus*, bringing the nanoparticles closer to the bacterial cell wall, thereby concentrating thermal energy and enhancing photothermal conversion efficiency. The photothermal effect generated by D-AuHNRs@CeO_2_ played a critical role in the antibacterial process, highlighting its considerable potential for reducing wound infection risk (Fig. S31).

Additionally, fluorescence imaging was employed to investigate the interaction between chiral materials with different configurations and bacteria (Fig. 4c and d). Fluorescein isothiocyanate (FITC)-labeled chiral nanomaterials were used to study their interaction with the bacterial cell wall. The results showed that upon co-incubation of the materials with bacteria, the green fluorescence emitted by D-AuHNRs@CeO_2_ overlapped significantly with the blue fluorescence of *S*. *aureus* and *E*. *coli* stained with 4′,6-diamidino-2-phenylindole (DAPI). This phenomenon indicated that D-AuHNRs@CeO_2_ could adhere to bacterial surfaces and penetrate bacterial cell walls. In contrast, although the green fluorescence intensity of L- and DL- AuHNRs@CeO_2_ did not diminish, their overlapping fluorescence with DAPI was relatively weak, with most of the materials remaining free outside the bacteria or attached to the outer walls, unable to effectively penetrate the bacterial cell wall. These observations suggested that D-AuHNRs@CeO_2_ exhibited stronger interaction with bacteria than L- and DL-AuHNRs@CeO_2_, and were more readily adsorbed onto and able to penetrate the bacterial cell wall. To unravel the mechanistic origin underlying the divergent antibacterial performances of D-/L-/DL-AuHNRs@CeO_2_, isothermal titration calorimetry (ITC) was applied to quantify the binding thermodynamics between the three chiral nanoconfigurations and *S. aureus* as well as *E. coli* (Fig. 4e–g and S32) [48], which revealed that D-AuHNRs@CeO_2_ yielded notably lower dissociation constants (K_d_, M) toward both bacterial strains relative to the L- and DL-type counterparts. This observation confirmed that the D-chiral surface of the nanoplatform mediates stereoselective recognition with bacterial surfaces, which ultimately translates into a substantially enhanced binding affinity.

Furthermore, the leakage of nucleic acids and proteins from *S*. *aureus* and *E*. *coli* was further quantified (Fig. 4h–k). The results indicated that chiral AuHNRs and AuHNRs@CeO_2_ treatments triggered the release of intracellular nucleic acids into the surrounding environment. In particular, the significantly increased nucleic acid leakage in *S. aureus* and *E. coli* treated with NIR + D-AuHNRs@CeO_2_ was consistent with the previous findings.

### 3.5 Cellular antioxidant, migration-promoting, and molecular regulatory mechanisms of chiral AuHNRs@CeO_2_

Building on the aforementioned research findings that the chiral AuHNRs@CeO_2_ composite nanomaterials exhibited significantly enhanced antibacterial efficacy and antioxidant activity compared to single-component chiral AuHNRs, these composites were selected for further investigation. Cytotoxicity testing was first performed on various cell lines with the 3-(4,5-dimethylthiazol-2-yl)-2,5-diphenyltetrazolium bromide (MTT) method. Based on the hemolysis results, the therapeutic concentration of the chiral nanomaterials was ultimately set at 100 μg/mL (Fig. S33). Next, to evaluate the antioxidant effect of chiral AuHNRs@CeO_2_ at the cellular level, this work employed 2′,7′-dichlorodihydrofluorescein diacetate (DCFH-DA) as a fluorescent probe, and then monitored the intracellular ROS levels in L929 fibroblasts, which were induced with H_2_O_2_ (Fig. S34) [49]. Results demonstrated that control cells treated with H_2_O_2_ exhibited strong green fluorescence signals, indicating a significant elevation in intracellular ROS levels. Conversely, cells co-treated with chiral AuHNRs@CeO_2_ and H_2_O_2_ showed markedly reduced fluorescence intensity, with the D-AuHNRs@CeO_2_ group exhibiting the weakest fluorescence, confirming the superior capacity of the D-form composite to scavenge excess intracellular ROS and mitigate oxidative stress damage (Fig. 5a and b).

**Fig. 5:**
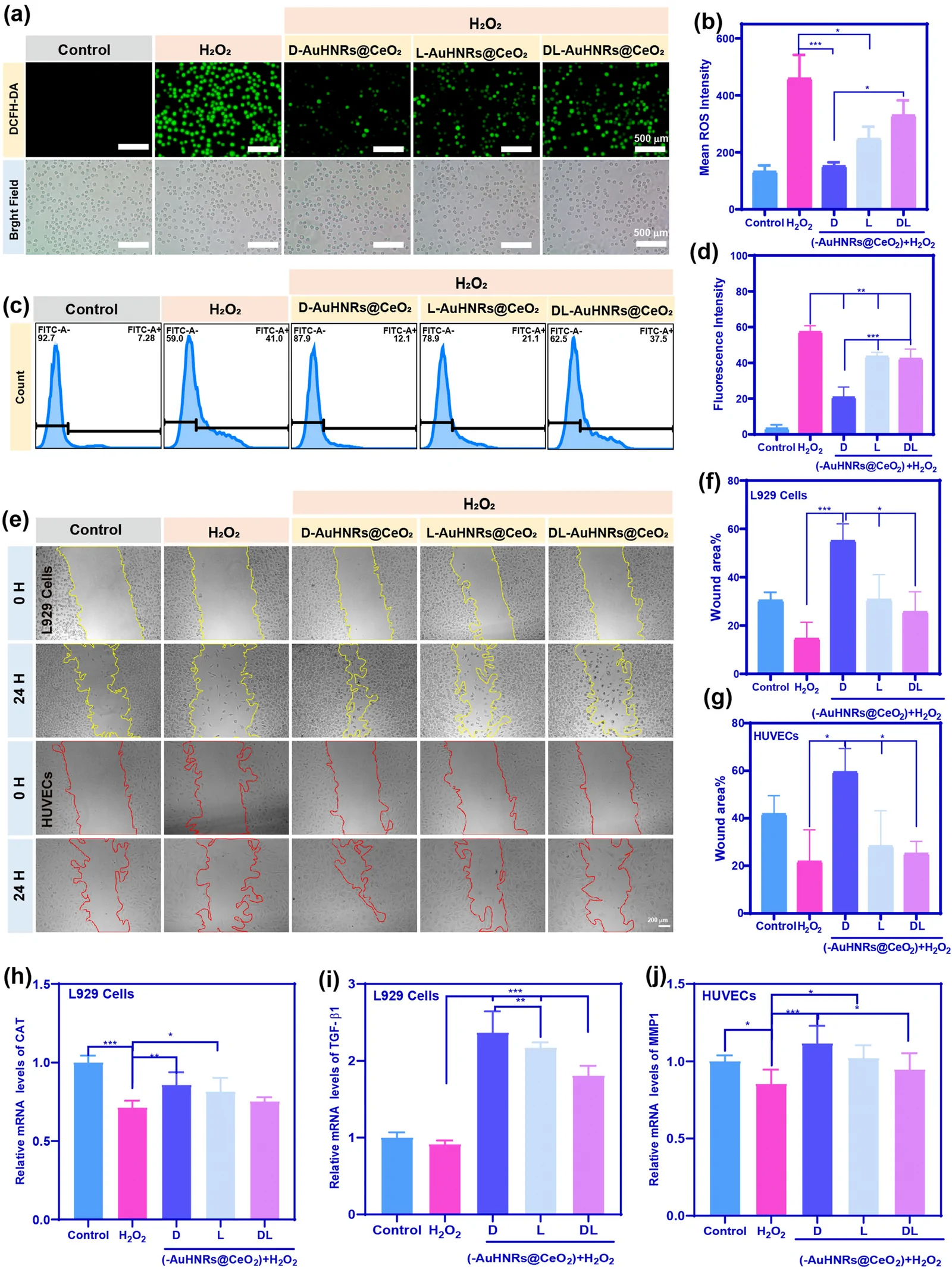
Antioxidant, anti-inflammatory, and pro-migratory activities of chiral AuHNRs@CeO_2_*in vitro*. (a) Representative fluorescent images of intracellular ROS in L929 cells stained with DCFH-DA after treatment with H_2_O_2_ alone or in combination with chiral nanomaterials (scale bar: 500 μm). (b) Statistical analysis of mean fluorescence intensity. (c) Flow cytometry analysis of L929 cells stained with DCFH-DA after various treatments. (d) Statistical quantification of DCFH-DA fluorescence intensity from flow cytometry. (e) Migration images of L929 cells and HUVECs after different treatments. (f) Migration rate of L929 cells. (g) Migration rate of HUVECs. Effects of different treatments on the mRNA expression levels of (h) CAT and (i) TGF-β1 in H_2_O_2_-stimulated L929 cells. Effects of different treatments on the mRNA expression levels of (j) MMP1 in H_2_O_2_-stimulated HUVECs.

To further corroborate these findings quantitatively, flow cytometry was employed to assess intracellular ROS levels. As expected, treatment with chiral AuHNRs@CeO_2_ significantly attenuated H_2_O_2_-induced oxidative stress damage in L929 cells. Specifically, the proportion of ROS-positive cells in the D-AuHNRs@CeO_2_ group was significantly lower than that in the L-AuHNRs@CeO_2_ and DL-AuHNRs@CeO_2_ groups. These results collectively demonstrated that the D-type composite material exhibited superior biological effects in scavenging intracellular ROS and alleviating oxidative stress damage (Fig. 5c and d).

Cell migration and angiogenesis serve as key indicators reflecting a nanomaterial's potential to promote wound healing [50]. To simulate an oxidative stress microenvironment, the effect of chiral AuHNRs@CeO_2_ on L929 cell migration under H_2_O_2_ stimulation was evaluated (Fig. 5e–g). These results revealed that after 24 h of co-culture with chiral AuHNRs@CeO_2_, the D-AuHNRs@CeO_2_ group exhibited a statistically significant increase in wound closure rate compared to the blank control group, whereas the L- and DL-AuHNRs@CeO_2_ groups showed relatively poor healing efficiency. Similarly, scratch assays performed with HUVECs yielded results highly consistent with the L929 cell experiments, further highlighting the superior pro-migratory effect of D-AuHNRs@CeO_2_.

To further elucidate the molecular mechanism underlying the pro-migratory effect of chiral AuHNRs@CeO_2_, the expression of relevant functional genes was examined in L929 and HUVEC cells following H_2_O_2_-induced oxidative stress. The results showed that D-AuHNRs@CeO_2_ treatment significantly upregulated the mRNA expression of CAT and TGF-β1 in L929 cells, as well as the expression of MMP1 in HUVECs (Fig. 5h–j). Without H_2_O_2_ stimulation, chiral AuHNRs@CeO_2_ exerted no obvious effects on the mRNA levels of CAT in L929 cells and MMP1 in HUVECs compared with the control group. Nevertheless, the mRNA expression of TGF-β1 was significantly upregulated in L929 cells (Fig. S35), suggesting that the chiral composite material possesses an intrinsic pro-repair property. In conclusion, these results are consistent with the previously demonstrated superior antioxidant and anti-inflammatory activities of D-AuHNRs@CeO_2_, indicating that the material exerts a synergistic effect by scavenging ROS and promoting cell migration, reflecting the synergistic advantage arising from the stereoselectivity of chiral AuHNRs and the functional loading of CeO_2_.

### 3.6 Cellular uptake and macrophage polarization by chiral AuHNRs@CeO_2_

Based on the above findings, uptake experiments were performed with FITC labeled chiral AuHNRs@CeO_2_, and nuclei were counterstained with DAPI. High-content microscopy imaging revealed that the fluorescence signal of D-AuHNRs@CeO_2_ exhibited a high degree of co-localization with the regions of L929 cells (DAPI-stained areas), indicating that the D-form material was engaged in close interaction with the cells. In contrast, the fluorescence signals of the L- and DL-AuHNRs@CeO_2_ groups showed significantly less co-localization, which was greatly confined to the periphery of the cell membrane (Fig. 6a). Importantly, under normal culture conditions, the fluorescence of all three chiral nanomaterials was predominantly located around the cells, suggesting that their internalization behavior was dependent on the oxidative stress state. These imaging observations implied that the D-AuHNRs@CeO_2_ might promote its adsorption or internalization in oxidative stressed cells, which could facilitate the delivery of its functional components to intracellular targets (Fig. 6b).

**Fig. 6:**
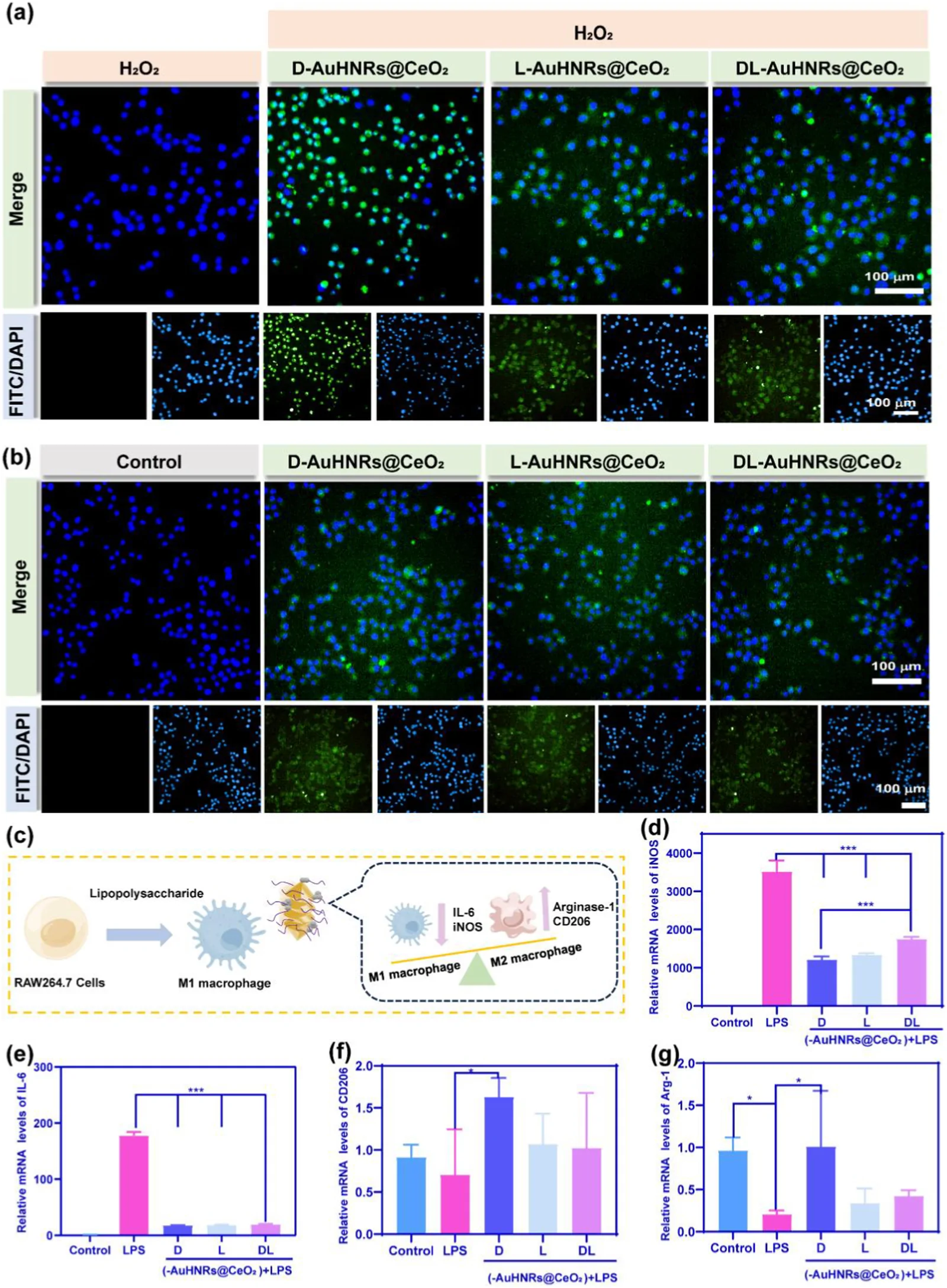
Cellular uptake and macrophage polarization by chiral AuHNRs@CeO_2_. (a) Interaction images between FITC labeled chiral AuHNRs@CeO_2_ and L929 cells under oxidative stress (scale bars: 100 μm). (b) Interaction images of L929 cells with chiral AuHNRs@CeO_2_ labeled with FITC (scale bar: 100 μm). (c) Schematic summary of D-AuHNRs@CeO_2_-induced M1-to-M2 macrophage polarization. Effects of different treatments on the mRNA expression levels of (d) iNOS, (e) IL-6, (f) CD206 and (g) Arg-1 in LPS -stimulated RAW264.7 cells. (***p < 0.001, **p < 0.01, *p < 0.05).

To further investigate whether this internalization endows D-AuHNRs@CeO_2_ with the ability to modulate macrophage function, lipopolysaccharide (LPS) stimulated RAW264.7 cells were employed [51]. The results confirmed that D-AuHNRs@CeO_2_ treatment significantly downregulated the M1-type markers (iNOS and IL-6) and upregulated the M2-type markers (CD206 and Arg-1, Fig. 6c–g). Therefore, these results demonstrated that the oxidative-stress-dependent uptake of D-AuHNRs@CeO_2_ facilitated its intracellular delivery, thereby enabling effective regulation of macrophage polarization toward a pro-repair phenotype.

These results confirmed that the cellular uptake of D-AuHNRs@CeO_2_ was oxidative-stress-dependent. Specifically, efficient internalization occurred only under oxidative stress, a behavior not observed with the L- or DL-type materials. D-AuHNRs@CeO_2_ promoted the polarization of macrophages toward the anti-inflammatory M2 phenotype, thereby creating a favorable microenvironment for the migration of fibroblasts and endothelial cells. Ultimately, a synergistic cascade was established, comprising efficient cellular uptake, enhanced ROS clearance, suppressed inflammation, and promoted cell migration.

### 3.7 *In vivo* wound healing and antibacterial activity of chiral AuHNRs@CeO_2_

Given the excellent *in vitro* antibacterial efficacy of chiral AuHNRs@CeO_2_, its therapeutic efficacy *in vivo* was further validated. For this purpose, *S*. *aureus*, the most frequently detected pathogen in clinical wound infections, was chosen as the infection model [52]. A full-thickness skin defect wound model was created in BALB/c mice to assess *in vivo* antibacterial activity and wound-healing promotion. After wound induction (day 0), all wounds presented remarkable swelling and exudation, confirming successful model establishment (Fig. 7a). Mice were then randomly allocated into eight treatment groups: control, control + NIR, D-AuHNRs@CeO_2_, D-AuHNRs@CeO_2_ +NIR, L-AuHNRs@CeO_2_, L-AuHNRs@CeO_2_+NIR, DL-AuHNRs@CeO_2_ and DL-AuHNRs@CeO_2_ + NIR.

**Fig. 7:**
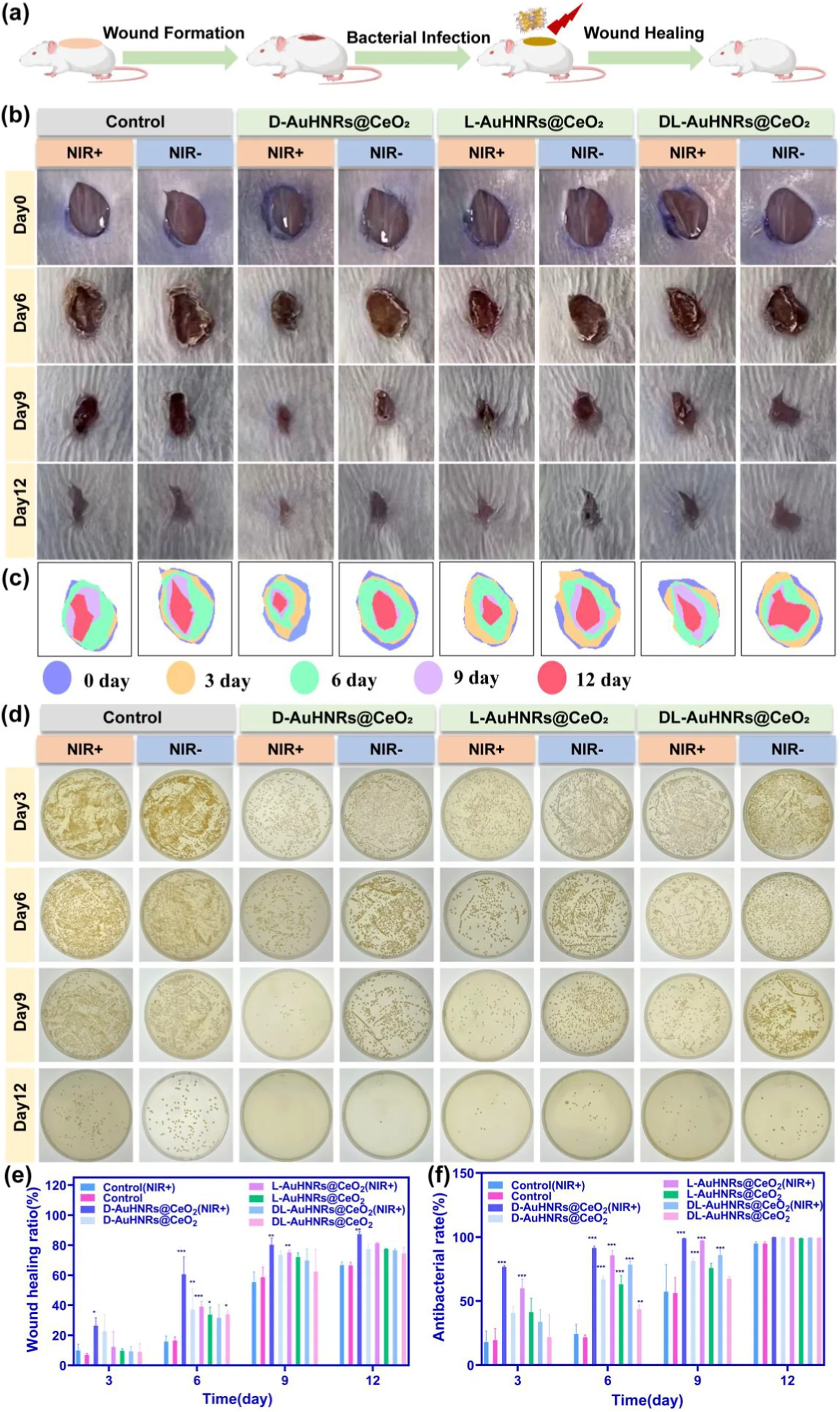
*In vivo* antibacterial activity of chiral AuHNRs@CeO_2_ against *S. aureus* infection in BALB/c mice. (a) Schematic of the cutaneous infection model and the various treatment regimens. (b) Schematic representation of the wound healing process over time for each treatment group. (c) Wound simulation diagrams for each group. (d) Bacterial colony images collected from wound sites of mice in each treatment group. (e) Quantitative assessment of wound closure rates across treatment groups. (f) Quantification of bacterial colony counts from wound sites (n = 5, ***p < 0.001, **p < 0.01, *p < 0.05).

After the respective treatments, wound healing was tracked and imaged at predefined time points (Fig. 7b). Healing rates were calculated and the process was mathematically modeled (Fig. 7c–e). Wounds treated with D-AuHNRs@CeO_2_ + NIR showed pronounced contraction and enhanced healing at 6, 9, and 12 days post-treatment. To quantitatively assess *in vivo* antibacterial activity, bacteria collected from the wound sites were cultured on agar plates on days 3, 6, 9, and 12 (Fig. 7d), and bacterial colonies were enumerated (Fig. 7f). Consistent with the *in vitro* findings, NIR-irradiated groups consistently showed stronger antibacterial effects than their non-irradiated counterparts did, whereas the other treatments displayed limited efficacy. These findings demonstrated that chiral AuHNRs@CeO_2_ under NIR irradiation could effectively eliminate *S*. *aureus* in the early stage of wound management. Notably, the D-AuHNRs@CeO_2_+NIR group yielded the most robust therapeutic outcome, with both the lowest bacterial loading and the most accelerated wound healing in mice. To further evaluate the broad-spectrum antibacterial potential of chiral AuHNRs@CeO_2_, an additional *E. coli* infection model was established in mice, using levofloxacin hydrochloride as the positive control (Fig. S36–S37). The D-AuHNRs@CeO_2_ + NIR group exhibited the most pronounced antibacterial effect among all tested groups, comparable to that of levofloxacin. These results collectively demonstrated that the chiral AuHNRs@CeO_2_ platform possessed broad-spectrum antibacterial efficacy against both Gram-positive and Gram-negative bacteria.

Body weight monitoring across all groups revealed only minor fluctuations and showed no significant weight loss or physiological distress, further supporting the good biocompatibility and low systemic toxicity of the chiral AuHNRs@CeO_2_ composite *in vivo* (Fig. S38–39).

### 3.8 Tissue regeneration and anti-inflammatory efficacy of chiral AuHNRs@CeO_2_

Histological evaluation of wound healing progression and inflammatory status in infected wounds was performed on days 6 and 12 using hematoxylin and eosin (H&E) and Masson's trichrome staining (Masson's staining). Control and control + NIR groups revealed severe inflammatory infiltration, characterized by abundant neutrophils and lymphocytes, as well as epidermal necrosis and detachment, reflecting substantial tissue damage that considerably impeded the healing process. By contrast, wounds treated with D-AuHNRs@CeO_2_ + NIR displayed a flattened wound surface and significantly fewer inflammatory cells, confirming their pronounced anti-inflammatory and wound-accelerating efficacy in infected wounds (Fig. 8a and b).

**Fig. 8:**
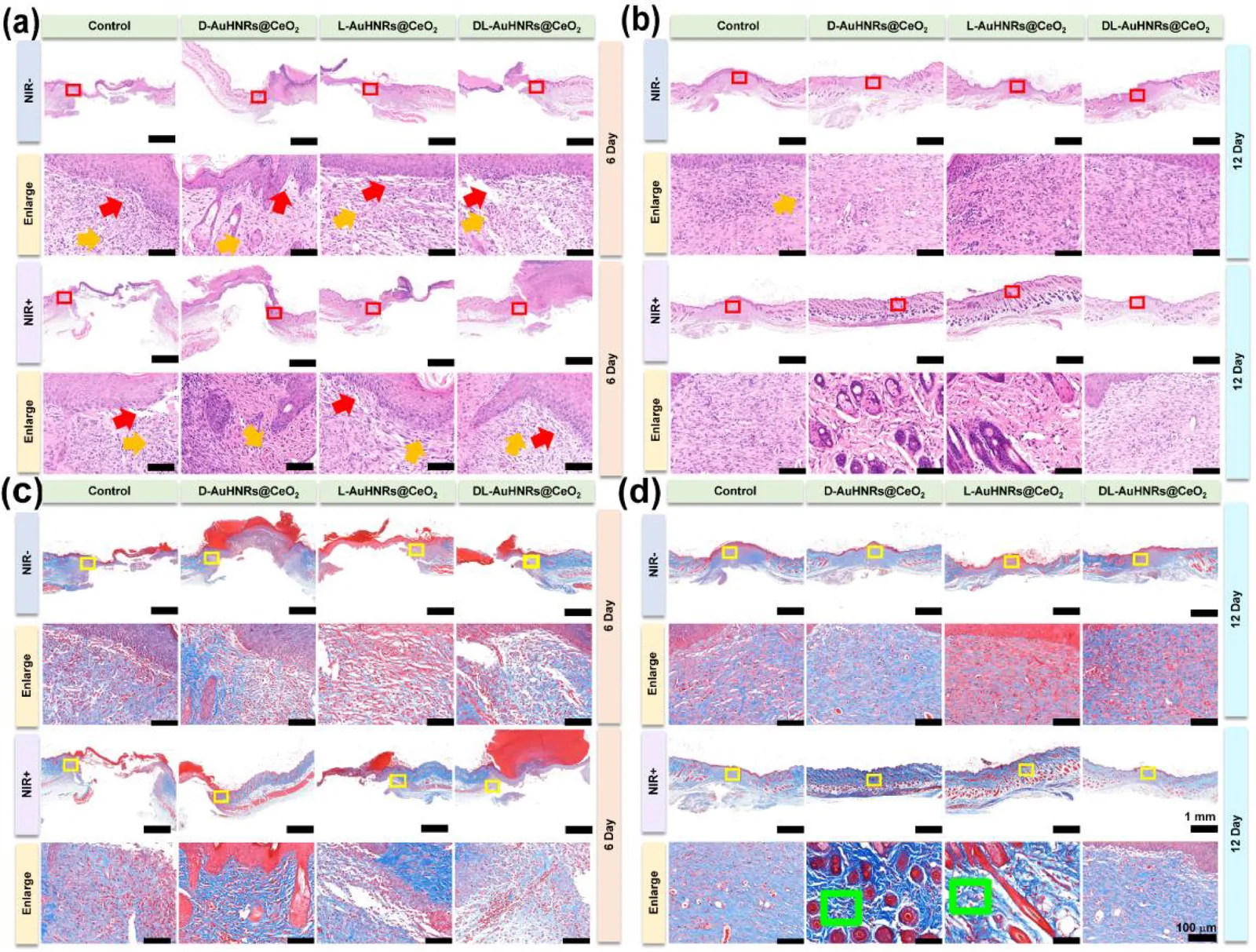
Histological analysis of wound tissues after treatment with chiral AuHNRs@CeO_2_. (a) Representative H&E-stained images of wound sections on day 6 (yellow arrows, inflammatory cells; red arrows, loose connective tissue). (b) Representative H&E-stained wound tissue sections images on day 12. (c) Representative Masson-stained wound tissue sections images on day 6. (d) Representative Masson-stained wound tissue sections images on day 12 (green rectangle, organized collagen fibers; Scale bar: above, 1 mm; below, 100 μm). (For interpretation of the references to color in this figure legend, the reader is referred to the Web version of this article.)

Masson's staining further corroborated the healing trends. Both the control and control + NIR groups presented loose connective tissue and disorganized collagen fibers, indicating severely compromised tissue regeneration. Conversely, wounds treated with D-AuHNRs@CeO_2_+ NIR displayed nascent granulation tissue, newly formed hair follicles and sebaceous glands, regenerated vasculature, and elongated fibroblasts at day 6, which demonstrated enhanced healing relative to other groups. These morphological features were consistent with actively promoted re-epithelialization (Fig. 8c). By day 12, the D-AuHNRs@CeO_2_ + NIR group showed abundant, densely packed, and well-aligned collagen fibers, a pattern markedly more advanced than in the control and control + NIR groups. Moreover, the D-AuHNRs@CeO_2_ + NIR group exhibited a nearly complete, relatively thick epidermis with high keratinocyte density (Fig. 8d). These findings aligned with H&E observations, confirming the superior wound-healing capacity of chiral AuHNRs@CeO_2_ under NIR irradiation, with the D-AuHNRs@CeO_2_ + NIR group showing the most pronounced efficacy. To assess potential organ toxicity, histopathological analysis was performed on major organs (heart, liver, spleen, lungs, and kidneys). No significant abnormalities or lesions were detected, indicating excellent biocompatibility and safety of the chiral nanomaterials and supporting their potential for future clinical translation (Fig. S40).

## 4 Conclusion

In summary, this work reports a class of chiral AuHNRs@CeO_2_ composites fabricated with D-Cys and L-Cys as chiral inducers, which integrate photothermal performance and multiple synergistic functions. *In vitro* investigations confirm that the embedded CeO_2_ synergizes well with the photothermal effect of chiral AuHNRs, achieving efficient elimination of *S. aureus* and *E. coli* as well as effective ROS scavenging to realize combined antibacterial and antioxidative effects. Systematic *in vivo* evaluations further verify its excellent biocompatibility: the optimized D-AuHNRs@CeO_2_ can penetrate cell membranes to clear excess intracellular ROS, promote fibroblast migration via upregulating key repair-related biomarkers (CAT, TGF-β1 and MMP1), and induce macrophage polarization from the pro-inflammatory M1 phenotype to the pro-healing M2 phenotype. In the BALB/c mouse model of infected wounds, D-AuHNRs@CeO_2_ significantly reduces local inflammatory infiltration, accelerates collagen deposition and neovascularization, and improves skin regeneration for faster wound closure. Notably, both antibacterial and antioxidant activities exhibit clear chirality dependence, where D-AuHNRs@CeO_2_ delivers remarkably superior performance in disrupting *S. aureus* structures and scavenging intracellular ROS compared with its L- and DL-counterparts, which is mainly attributed to the stronger stereoselective interactions between D-type nanomaterials and bacterial membranes or cellular components. Overall, this chiral AuHNRs@CeO_2_ system adopts physical sterilization as the core modality and microenvironment regulation as the auxiliary strategy, and by combining the synergistic triad of “photothermal, antioxidant and anti-inflammatory” effects with chirality-dependent targeting, it provides a novel and promising therapeutic route for the precise treatment of bacterial wound infections.

## CRediT authorship contribution statement

**Jinglei Xu:** Writing – original draft, Validation, Methodology, Investigation, Data curation, Conceptualization. **Qiusha Li:** Investigation, Formal analysis, Data curation. **Wen Kan:** Visualization, Investigation, Formal analysis. **Wuyi Liu:** Methodology, Investigation. **Chen Liu:** Methodology, Investigation, Conceptualization. **Qiuyue Liu:** Investigation, Formal analysis. **Jie Gao:** Methodology, Conceptualization. **Kaichuan Yan:** Writing – original draft, Methodology, Conceptualization. **Chengzhi Huang:** Writing – review & editing, Supervision, Project administration, Conceptualization. **Jian Wang:** Writing – review & editing, Supervision, Project administration, Funding acquisition, Conceptualization.

## Ethics statement

Female BALB/c mice (6–8 weeks old) were purchased from Hunan Silek Jingda Experimental Animal Co., Ltd. All animals were housed in specific pathogen-free (SPF) facilities. All animal procedures were reviewed and approved by the Institutional Animal Care and Use Committee (IACUC) of Southwest University (Approval No. IACUC-20251222-02).

## Declaration of competing interests

The authors declare that they have no known competing financial interests or personal relationships that could have appeared to influence the work reported in this paper.

## Data Availability

Data will be made available on request.

## References

[1] Zhang W., Lu H., Zhang W., Hu J., Zeng Y., Hu H., Shi L., Xia J., Xu F. (2024). Inflammatory microenvironment‐responsive hydrogels enclosed with quorum sensing inhibitor for treating post‐traumatic osteomyelitis. Adv. Sci..

[2] Lin X., Zhang X., Wang Y., Chen W., Zhu Z., Wang S. (2025). Hydrogels and Hydrogel-Based drug delivery systems for promoting refractory wound healing: applications and prospects. Int. J. Biol. Macromol..

[3] She Y., Wu P., Wan W., Liu H., Liu R., Wang T., Wang M., Shen L., Yang Y., Huang X. (2025). Polysaccharides, proteins and DNA based stimulus responsive hydrogels promoting wound healing and repair: a review. Int. J. Biol. Macromol..

[4] Wei Q., Fan Z., Chen H., Zhang W., Jiang Y., Hu Z., Wang Q. (2025). A nanocomposite Xanthan Gum/Puerarin supramolecular hydrogel with immunoregulatory, antibacterial, and antioxidant properties for wound healing. Carbohydr. Polym..

[5] Mo D., Pan M., Chen W., Liu Q., Yu Y., Yuan L., Yang Y., Deng H., Wang M., Qian Z. (2024). Sulfur vacancy‐rich Bi_2_S_3_‐X@PDA heterojunctions with light‐controlled reactive oxygen species generation and elimination to combat biofilm infection and inflammation caused by drug‐resistant bacteria. Adv. Funct. Mater..

[6] Cui X., Ruan Q., Zhuo X., Xia X., Hu J., Fu R., Li Y., Wang J., Xu H. (2023). Photothermal nanomaterials: a powerful light-to-heat converter. Chem. Rev..

[7] Xie M., Gao M., Yun Y., Malmsten M., Rotello V.M., Zboril R., Akhavan O., Kraskouski A., Amalraj J., Cai X. (2023). Antibacterial nanomaterials: mechanisms, impacts on antimicrobial resistance and design principles. Angew. Chem., Int. Ed..

[8] Makabenta J.M.V., Nabawy A., Li C.-H., Schmidt-Malan S., Patel R., Rotello V.M. (2021). Nanomaterial-Based therapeutics for antibiotic-resistant bacterial infections. Nat. Rev. Microbiol..

[9] Li L.T., Feng Y.S., Liu C., Xiong Y.Y., Shi H.F., Liu Q.Y., Li Q.S., Zhao X.H., Wang J. (2025). Single-Stranded nucleic acid-induced aggregation of intrinsically chiral helical gold nanorods for biosensing applications. Anal. Chem..

[10] Wang G., Hao C., Ma W., Qu A., Chen C., Xu J., Xu C., Kuang H., Xu L. (2021). Chiral plasmonic triangular nanorings with SERS activity for ultrasensitive detection of amyloid proteins in Alzheimer's Disease. Adv. Mater..

[11] Wen S., Shi H.F., Zhao C.N., Xiong Y.Y., Li L.T., Liu Q.Y., Liu C., Li Q.S., Wang W., Wang J. (2025). Structure-Optical activity relationship of chiral helical gold nanorods and their application in alkaline phosphatase detection. Chem. Eng. J..

[12] Luo J., Shi B., Hao C., Sun M., Xu L., Kuang H., Qu A. (2025). Chiral zinc sulfide nanoparticles scavenging reactive oxygen species for remodeling intestinal homeostasis. Angew. Chem., Int. Ed..

[13] Zhang Y., Cui T., Yang J., Huang Y., Ren J., Qu X. (2023). Chirality‐Dependent reprogramming of macrophages by chiral nanozymes. Angew. Chem., Int. Ed..

[14] Han J., Liu H., Chen J., Jia K., Sun J., Nie Z. (2024). Chirality-Dependent reprogramming of macrophages. ACS Nano.

[15] Zhao H., Jiao J., Wang Z., Shi C., Huang X., Lin Y., Yang H., Lei H., Ouyang G., Liu M. (2026). Amino acid directed helical assemblies of natural berberine for chirality‐dependent photodynamic antibacterial therapy. Adv. Sci..

[16] Zheng H., Wang X., Gao Z., Zhang X., Liu J., Yan J., Li N., Chen R., Xu Y., Yang Y., Chen C., Niu M., Jiang Y., Jiang S., Wei Y. (2025). Chiral gold nanoparticle synergizes Immunometabolic Crosstalk and metabolism remodeling for diabetic wound healing. Biomaterials.

[17] Wang W., Hao C., Sun M., Xu L., Wu X., Xu C., Kuang H. (2018). Peptide mediated chiral inorganic nanomaterials for combating gram‐negative bacteria. Adv. Funct. Mater..

[18] Wang G., Hao C., Chen C., Kuang H., Xu C., Xu L. (2022). Six‐Pointed Star chiral cobalt superstructures with strong antibacterial activity. Small.

[19] Niu X., Zhao R., Yan S., Pang Z., Li H., Yang X., Wang K. (2023). Chiral materials: progress, applications, and prospects. Small.

[20] Song W., Wang X., Nong S., Wang M., Kang S., Wang F., Xu L. (2024). D‐Cysteine‐Derived carbon dots for selective discrimination, imaging, and synergistic elimination of gram‐positive bacteria and fungi. Adv. Funct. Mater..

[21] Huang X., Lu C., Zhang W., Liu L., Zha Z., Miao Z. (2023). Chiral Sulfur nanosheets for dual-selective inhibition of gram-positive bacteria. ACS Nano.

[22] Wang Z., Tian Y., Hao J., Liu Y., Tang J., Xu Z., Liu Y., Tang B., Huang X., Zhu N. (2024). Chiral nanoclusters as alternative therapeutic strategies to confront the health threat from antibiotic-resistant pathogens. ACS Nano.

[23] Zhang M., Zhang H., Feng J., Zhou Y., Wang B. (2020). Synergistic chemotherapy, physiotherapy and photothermal therapy against bacterial and biofilms infections through construction of chiral Glutamic Acid functionalized gold nanobipyramids. Chem. Eng. J..

[24] Chen P., Wang G., Hao C., Ma W., Xu L., Kuang H., Xu C., Sun M. (2022). Peptide-Directed synthesis of chiral nano-bipyramids for controllable antibacterial application. Chem. Rev..

[25] Huang X., Chen Q., Ma Y., Huang C., Zhi W., Li J., Zeng R., Pi J., Xu J.-f., Xu J. (2024). Chiral Au Nanostars for Sers Sensing of Enantiomers Discrimination, Multibacteria Recognition and Photothermal Antibacterial Application. Chem. Eng. J..

[26] Du Y., Gu Y., Wang W., Jiang Y., Fang X., Li Z., Niu L., Zhao L. (2024). Chiral ligands and photothermal synergistic effects of inorganic nanoparticles for bacteria-killing. J. Colloid Interface Sci..

[27] Jiang Y., Gou Y., Shen H., Wang Y., Zhang W., Wu Y., Liu L., Fang S., Hu M., Wang W., Xu L., Qian H. (2026). Chessboard microneedle array for inflammation regulation via spatiotemporally controlled drug delivery. Adv Mater.

[28] Zhao Y., Zhao S., Du Y., Gao Z., Li Y., Ma H., Li H., Ren X., Fan Q., Wu D. (2025). Inverse Oxide/Alloy‐Structured nanozymes with NIR‐Triggered enzymatic Cascade regulation of ROS homeostasis for efficient wound healing. Adv. Mater..

[29] Zhou B., Duan Y., Li W., Chen T., Wang J., Cao M., Lin G., Yang K., Lai Z., Wu W. (2025). A tailored hydrogel with local glycemia management, antioxidant activity, and photothermal antibacterial properties for diabetic wound healing. Adv. Sci..

[30] Zhou X., Zhou Q., He Z., Xiao Y., Liu Y., Huang Z., Sun Y., Wang J., Zhao Z., Liu X. (2024). ROS balance autoregulating core-shell CeO_2_@ZIF-8/Au nanoplatform for wound repair. Nano-Micro Lett..

[31] Ge L., Ling J., Cao G., kun Ouyang X., Wang N. (2025). Nanozyme doped Chitosan Hydrogel driven by glucose oxidase for dual regulation of ROS homeostasis and macrophage reprogramming in diabetic wound healing. Carbohydr. Polym..

[32] Hu M., Chen X., Lv X., Sui R., He R., Wu W., Zhu Y., Zhou J., Duan J., Cheng H., Chen W., Ma H. (2026). Bilirubin Co-Loaded CeO_2_ nanoparticles mitigate sepsis-related encephalopathy via synergistic anti-inflammation and neuroprotection. J. Control. Release.

[33] Ger T.-Y., Mousavi S.M., Yang C.-J., Yao C.-H., Lai J.-Y. (2026). Engineered cerium oxide nanoparticles conjugated with carrier platforms: therapeutic mechanisms for skin wound healing applications. Acta Biomater..

[34] Jiang P., Zhang L., Liu X., Ye C., Zhu P., Tan T., Wang D., Wang Y. (2024). Tuning oxidant and antioxidant activities of Ceria by anchoring copper single-site for antibacterial application. Nat. Commun..

[35] Wang Y., Liu K., Wei W., Dai H. (2024). A multifunctional hydrogel with photothermal antibacterial and antioxidant activity for smart monitoring and promotion of diabetic wound healing. Adv. Funct. Mater..

[36] Wang F., Yang W., Ding Q., Xing X., Xu L., Lin H., Xu C., Li S. (2025). Chiral Au@CeO_2_ helical nanorods with spatially separated structures for polarization-dependent N_2_ photofixation. Angew. Chem., Int. Ed..

[37] Zhang X., Yang P., Liu Y., Pan J., Li D., Wang B., Feng J. (2020). Support morphology effect on the selective oxidation of glycerol over AuPt/CeO_2_ catalysts. J. Catal..

[38] Xu X., Shao C., Zhang J., Wang Z., Dai K. (2024). Rational design of S-Scheme CeO_2_/Bi_2_MoO_6_ microsphere heterojunction for efficient photocatalytic CO_2_ reduction. Acta Phys. Chim. Sin..

[39] Liu K., Zhu H., Shi F., Li J., Li X., Lai Z., Li H., Zeng H., Yang Z., Pang H. (2025). Three‐Channel electron engineered Fe‐88A@CeO_2_/CDs nanozyme with enhanced oxidase‐like activity for efficient biomimetic catalysis. Adv. Sci..

[40] Wang X., Qin X., Liu Y., Fang Y., Meng H., Shen M., Liu L., Huan W., Tian J., Yang Y.W. (2024). Plasmonic Supramolecular nanozyme‐based Bio‐Cockleburs for synergistic therapy of infected diabetic wounds. Adv. Mater..

[41] Xiao K., Cao Y., Han Z., Zhang Y., Luu L.D.W., Chen L., Yan P., Chen W., Wang J., Liang Y. (2025). A pan-immune panorama of bacterial pneumonia revealed by A large-scale single-cell transcriptome Atlas. Signal Transduct. Targeted Ther..

[42] Mylonas A., Hawerkamp H.C., Wang Y., Chen J., Messina F., Demaria O., Meller S., Homey B., Di Domizio J., Mazzolai L. (2023). Type I IFNs link skin-associated dysbiotic Commensal bacteria to pathogenic inflammation and angiogenesis in Rosacea. JCI Insight.

[43] Zhang Q., Yu B.X., Long J., Chen X.W., Huang F., Wu W.Q., Liu Y.D., Zeng G.H., Huang L.H., Sun X.Y. (2025). An Ultrasmall self‐assembled gallic‐acid‐based natural multifunctional defense networks for therapeutic application in calcium oxalate nephropathies. Small.

[44] Reznickova A., Slavikova N., Kolska Z., Kolarova K., Belinova T., Kalbacova M.H., Cieslar M., Svorcik V. (2019). PEGylated gold nanoparticles: stability, cytotoxicity and antibacterial activity. Colloids Surf., A.

[45] Zhang B., Chen Y., Hou S., Liu Z., Yang Y., Li T., Zhao L., Zhang H., Wei H., Meng L., Jing X., Guan H. (2026). Smart microenvironment-adaptive nanocatalytic Hydrogel for sequential antibacterial, Anti-Inflammatory, and regenerative therapy of biofilm-infected wounds. Bioact. Mater..

[46] Pütz E., Gazanis A., Keltsch N.G., Jegel O., Pfitzner F., Heermann R., Ternes T.A., Tremel W. (2022). Communication breakdown: into the molecular mechanism of biofilm inhibition by CeO_2_ nanocrystal enzyme mimics and how it can be exploited. ACS Nano.

[47] Nosrati H., Heydari M., Khodaei M. (2023). Cerium oxide nanoparticles: synthesis methods and applications in wound healing, Mater. Today Bio.

[48] Zhao K., Hou L., Li F., Xue Y., Jin Y., Li B. (2026). Boosting antibacterial efficacy of silicon Quantum dots through chiral surface engineering. ACS Appl. Mater. Interfaces.

[49] Wang H., Sun S., Zhao Y., Wang P., Zhou Y., Sun H., Yang J., Cheng K., Li S., Lin H. (2024). Carbon dots with integrated photothermal antibacterial and heat‐enhanced antioxidant properties for diabetic wound healing. Small.

[50] Guo Q., Zhang M., Zheng J., Li Z., Zhang Y., Chen Y., Chen Y., Jiang X., Tang J. (2025). A multifunctional MXene@CeO_2_‐Enhanced hydrogel dressing for synergistic photothermal antibacterial and antioxidative therapy in wound healing. Adv. Healthcare Mater..

[51] Tian X., Wen Y., Zhang Z., Zhou K., Shang L., Zhu J., Song X., Li J. (2026). Smart multifunctional ROS-Responsive supramolecular hydrogel for simultaneously regulating oxidative stress, immune dysregulation, and bacterial infection in diabetic wound healing. Biomaterials.

[52] Giudice P.D. (2020). Skin infections caused by Staphylococcus aureus. Acta Derm. Venereol..

