# Supplementary material for: Synergistic photothermal and antioxidant effects of chiral gold helical nanorods @CeO2 nanomaterials for accelerated infected wound healing
Source: Mater Today Bio. 2026 Oct 15;41:103754. doi: 10.1016/j.mtbio.2026.103754 (PMC13698979; doi:10.1016/j.mtbio.2026.103754)
Supplement: Supplementary file 1 [file mmc1.docx]

Supplemental Materials for

Synergistic photothermal and antioxidant effects of chiral gold helical nanorods @CeO_2_ nanomaterials for accelerated infected wound healing

Jinglei Xu,^a^ Qiusha Li,^a^ Wen Kan,^b^ Wuyi Liu,^a^ Chen Liu,^a^ Qiuyue Liu,^a^ Jie Gao,^c^ Kaichuan Yan,^*, a^ Chengzhi Huang, ^*, a^ Jian Wang^*, a^

^a^ Key Laboratory of Luminescence Analysis and Molecular Sensing (Southwest University), Ministry of Education, Key Laboratory of Biomedical Analytics (Southwest University), Chongqing Science and Technology Bureau, College of Pharmaceutical Sciences, Southwest University, Chongqing 400715, P. R. China

^b^ Key Laboratory of New Drug Screening from Traditional Chinese Medicine, College of Pharmaceutical Sciences, Southwest University, Chongqing, 400715, P.R. China

^c^ Department of biological and chemical Engineering, Chongqing University of Education, Chongqing 400067, P. R. China

***Corresponding authors:**

Kaichuan Yan, Email: yankaichuan@foxmail.com; Chengzhi Huang, Email: chengzhi@swu.edu.cn, Jian Wang, Email: wj123456@swu.edu.cn

Experimental Sections

Reagents

Ascorbic acid (AA, ≥99.7%) was sourced from Chengdu Jinshan Chemical Reagent Co., Ltd. L-cysteine (L-Cys, 99%), D-cysteine (D-Cys, 98%), DL-cysteine (DL-Cys, 97%), tetrachloroauric(III) acid trihydrate (HAuCl₄·3H₂O), anhydrous cerium(III) acetate (Ce(Ac)₃), potassium tetrachloroplatinate(II) (K₂PtCl₄), and methoxypoly(ethylene glycol) thiol (mPEG-SH) were supplied by Shanghai Macklin Biochemical Technology Co., Ltd. Cetyltrimethylammonium bromide (CTAB) was obtained from Genview (Beijing, China). Hydrochloric acid (HCl) was procured from Chuandong Chemical Industry (Chongqing, China). Fluorescein PEG thiol (FITC-PEG-SH) was acquired from Shanghai Ponsure Biotech Inc. Silver nitrate (AgNO₃) was provided by Chron Chemicals (Sichuan, China). The live/dead bacterial viability kit containing N,N-dimethylaniline N-oxide (DMAO) and propidium iodide (PI) was purchased from Shanghai Beyotime Biotechnology Co., Ltd. 3,3’-Diethyloxacarbocyanine (DiOC₂(3)) was obtained from Beijing Solarbio Science and Technology Co., Ltd. Delivector™ Avertin was sourced from Dowobio (Shanghai, China). Tissue fixative was procured from Servicebio Technology Co., Ltd. All aqueous solutions were prepared with deionized water with a resistivity of 18 MΩ·cm.

**Characterization**

CD spectra were recorded with a J810 circular dichroism spectrometer (JASCO, Japan). Extinction spectra were acquired with a UV-2550 UV-vis spectrophotometer (Shimadzu, Japan). X-ray photoelectron spectroscopy (XPS) was measured using the XPS instrument (Thermo escalab 250XI, USA) with a radiation of 150 W Al Kα. Furthermore, morphologies of gold nanomaterials were characterized by scanning electron microscopy (SEM) using an S-4800 instrument (Hitachi, Japan) and high-resolution transmission electron microscopy (HRTEM, FEI, USA). Reaction conditions were maintained with an SHZ-82 constant temperature shaker (Lichen, China). An H1650W high-speed centrifuge (Hunan Xiangyi Company, China) was conducted to purify samples and an HCJ-6D water bath digital magnetic stirrer with water bath (Changzhou Longyue Company, China) was for controlling temperature during the experiments. The flow cytometry data was obtained by BD LSRFortessa Cell Analyzer. All the photos were captured from High-Content Screening (HCS). All RT-qPCR reactions were performed with a Toptical 96 Real-Time PCR System (Analytikjena, Germany). The 3D bacterial biofilm was examined using a Nikon super-resolution structured illumination microscopy (SIM) system (N-SIM E, Japan).

**Preparation of gold nanorods**

**Synthesis of Au seeds**: In a 25 mL clean round-bottom flask, 0.3645 g of CTAB was completely dissolved in 5 mL deionized water (18.2 MΩcm) water at 50 ℃ in a water bath, which was then allowed to cool naturally to ambient temperature. Subsequently, 5 mL of freshly prepared HAuCl_4_ solution (0.5 mM) was introduced into the CTAB solution with complete mixing. Under vigorous magnetic stirring (1200 rpm), 1 mL of ice-cold NaBH_4_ solution (6 mM, stored at 4 ℃) was added by dropwise (0.5 mL/min) using a micropipette, followed by continuous stirring for 2 min to complete the reduction reaction. The resulting solution was aged at 30 ℃ for 2 h in the dark.

To prepare gold nanorods (AuNRs), growth solution preparation was obtained by the following protocol [1]: In a 250 mL clean conical flask, 2.8000 g of CTAB and 0.4936 g of sodium oleate (NaOL) were dissolved in 95 mL of deionized water at 50 ℃ for 30 min with magnetic stirring (500 rpm) to complete dissolution. After cooling down to room temperature, AgNO_3_ solution (4 mM) with various volumes was added to control nanorod aspect ratios. The mixture was protected from light for 15 min, followed by the addition of 95 mL of freshly prepared HAuCl_4_ solution (1 mM), which was stirred (500 rpm) at 30 ℃ for 90 min in the dark to allow seed-mediated growth. Next, varying volumes of concentrated HCl (12 M) were then added, and stirring continued at 350 rpm for 15 min.

**AuNRs growth**: 0.25 mL of freshly prepared AA solution (64 mM) was injected rapidly into the growth solution. Immediate color change from orange to colorless was observed, indicating Au^3^⁺ reduction to Au⁺. Then, vigorous magnetic stirring (1200 rpm) was maintained for 30 s to ensure homogeneous mixing, followed by the introduction of predetermined volumes of aged gold seed solution for controlled anisotropic growth. Finally, the resulting solution was allowed to stand at 30 ^o^C for 12 h.

**Synthesis of chiral gold helical nanorods**

To prepare chiral gold helical nanorods (AuHNRs) [2], AuNRs solution was centrifuged at 8000 rpm for 8 min and redispersed in water. Then, 2.03 mL of AuNRs, 0.2 mL of CTAB (200 mM) and 40 μL of DL, L, D-Cys solution (6 mM) were combined and brought to a final volume of 4 mL with water. This mixture was incubated at 37 °C for 5 h. Subsequently, 40 μL of 7 mM AgNO_3_ solution was introduced to the above solution. After mixing evenly, 40 μL of 13 mM HAuCl_4_ and 20 μL of 100 mM AA solution were mixed with the above solution, which was heated immediately in a 70 °C water bath in the dark for 1 h.

**Synthesis of chiral AuHNRs@CeO_2_**

To synthesize chiral AuHNRs@CeO_2_ with an LSPR peak at 808 nm [3], 12 mL previously obtained AuHNRs was centrifuged and dispersed in 5 mL of 0.1 mM CTAB solution. Then, 0.24 mL of 0.1 mM K_2_PtCl_4_ solution was added and then stirred at 300 rpm and kept at room temperature for 4 min. After that, 0.12 mL of 10 mM Ce(AC)_3_ solution and 11.64 mL of ultrapure water were added to the prepared solution under continuous stirring at 300 rpm. The solution was heated at 100 °C for 1 h to form chiral AuHNRs@CeO_2_. The product was washed with ultrapure water for further use.

**Preparation of chiral AuHNRs (mPEG-SH) and AuHNRs@CeO_2_ (mPEG-SH)**

The chiral AuHNRs and AuHNRs@CeO_2_ were incubated in a 3 mM mPEG-SH solution prepared in 1× PBS containing 0.05% (v/v) Tween-20 at room temperature for 4 h to remove the CTAB adsorbed on the surface. After incubation, the mixtures were centrifuged at 6000 rpm for 4 min. The pellets were washed and redispersed in PBS to obtain mPEG‑SH‑modified AuHNRs and AuHNRs@CeO_2_.

**Photothermal effect**

The effect of chiral AuHNRs and AuHNRs@CeO_2_ concentration and laser power density on the photothermal conversion ability was investigated. Initially, chiral AuHNRs and AuHNRs@CeO_2_ suspensions with varying concentrations (0, 50, 100, 150 and 200 µg/mL) were continuously irradiated for 10 min (808 nm, 1.0 W/cm^2^). The temperature fluctuations were recorded every 30 s during these processes. Subsequently, the temperature of the chiral AuHNRs and AuHNRs@CeO_2_ suspension (100 µg/mL) upon 808 nm laser irradiation with varying power densities (0, 0.4, 0.6, 0.8, and 1.2 W/cm^2^) for 10 min was recorded.

**Photothermal stability**

The photothermal stability of chiral AuHNRs and AuHNRs@CeO_2_ was evaluated by subjecting their suspensions to five laser on-off cycles. Briefly, in each cycle, each suspension was irradiated with an 808 nm laser (0.8 W/cm^2^, 10 min) and then allowed to cool to ambient temperature for 10 min.

Calculation of the photothermal conversion efficiency (η): Chiral AuHNRs and AuHNRs@CeO_2_ suspensions (100 µg/mL) were irradiated with an 808 nm laser (1.0 W/cm^2^) for 10 min and then allowed to cool naturally.[4] The temperature was recorded every 30 s. The photothermal conversion efficiency (η) was calculated using the following formula and derivation process.

$$\text{η=}\frac{\text{hs}\text{(}\text{T}\text{max}\text{-T}\text{0}\text{)}}{\text{I(1-10}\text{-A}\text{808}\text{)}}\times100\%$$

where *T_max_* and *T_0_* are the maximum temperature of the sample and the ambient temperature, *I* is the incident laser power density (1.0 W/cm^2^ at 808 nm), A is the absorbance of the chiral AuHNRs (or chiral AuHNRs@CeO_2_) suspension at 808 nm, *h* is the heat transfer coefficient, and *S* is the surface area of the container.

$$\text{τ}\text{s}\text{=}\frac{\text{m×C}}{\text{hs}}$$

m = 1 g, C = 4.2 J g⁻¹

t = -τₛ(lnθ)

$$\text{θ=}\frac{\text{T-T}\text{surr}}{\text{T}\text{max}\text{-T}\text{surr}}$$

Here, *m* and *C* are the mass and heat capacity of water, respectively. 𝜏_s_ is the time constant obtained from the cooling curve. *T* is the temperature during cooling. *T_max_* and *T_surr_* are the maximum steady-state temperature of the nanoparticle suspension under irradiation and the ambient temperature of the surroundings, respectively.

**DPPH· detection method**

The antioxidant activity of chiral nanomaterials was evaluated using the 2,2-diphenyl-1-picrylhydrazyl (DPPH) free radical scavenging assay. Different final concentrations (150, 75, 37.5, 18.75, and 9.375 μg/mL) of chiral AuHNRs and AuHNRs@CeO_2_ were prepared. For each concentration, 300 μL of the nanomaterial suspension was mixed with 300 μL of a 0.2 mM DPPH ethanolic solution in a 1.5 mL centrifuge tube. The mixture was vortexed and incubated in the dark at 37 °C for 30 min. After incubation, the mixture was centrifuged at 8000 rpm for 5 min to precipitate the nanoparticles. Then, 200 μL of the supernatant was transferred to a 96‑well plate, and its absorbance was measured at 517 nm using a microplate reader, recorded as A__sample_. A control was prepared by replacing the DPPH solution with an equal volume of absolute ethanol, processed identically, and its absorbance was recorded as A__control_. The absorbance of the DPPH solution alone (mixed with ethanol) was measured as A__DPPH_. All experiments were performed in triplicate. The formula for calculating the DPPH scavenging rate is DPPH radical scavenging rate (%) = [(A__DPPH_ − (A__sample_ − A__control_)) / A__DPPH_ ] × 100%

**ABTS·⁺ detection method**

For the 2,2′-azino-bis(3-ethylbenzothiazoline-6-sulfonic acid) (ABTS) assay, the stock solution was diluted with ethanol to prepare a working solution with an absorbance of 0.70 ± 0.02 at 734 nm. Different final concentrations (150, 75, 37.5, 18.75, 9.375 μg/mL) of chiral AuHNRs and AuHNRs@CeO_2_ were prepared. For each sample, 300 μL of the nanomaterial suspension was mixed with 300 μL of the ABTS⁺· working solution and incubated at room temperature in the dark for 5 minutes. The mixture was then centrifuged at 8000 rpm for 5 min. The absorbance of the supernatant was measured at 734 nm and recorded as A__sample_. A sample background control was prepared by replacing the ABTS⁺· solution with an equal volume of PBS (or solvent), and its absorbance was recorded as A__control_. The absorbance of the ABTS⁺· working solution alone (mixed with PBS/solvent) was measured as A*__ABTS_*. All experiments were performed in triplicate.The ABTS⁺· radical scavenging efficiency was calculated as follows:Scavenging efficiency (%) = [ (A__ABTS_ − (A__sample_ − A__control_)) / A__ABTS_ ] × 100%

**·OH detection method**

·OH radical scavenging assay (TMB method): The hydroxyl radical (·OH) scavenging activity was evaluated using a TMB colorimetric method based on the Fenton reaction. Briefly, solutions of chiral AuHNRs and chiral AuHNRs@CeO_2_ at varying concentrations were mixed with 125 μM TMB and an equal volume of Fenton reagent (1 mM Fe^2^⁺/10 mM H_2_O_2_) in NaAc buffer (pH 4.5). After incubation at room temperature for 5 min, the absorbance at 652 nm was measured (A__sample_). Three controls were included: (1) Positive control: TMB + Fenton reagent (without nanomaterials), giving A__Fenton_. (2) Sample background control: Nanomaterials + TMB (without Fenton reagent), giving A__control_. (3) Reagent blank: Buffer only. The ·OH scavenging rate was calculated after background correction: Scavenging rate (%) = [ (A__Fenton_ − (A__sample_ − A__control_)) / A__Fenton_ ] × 100%

·OH radical scavenging assay (MB method): The scavenging activity was assessed by measuring the inhibition of methylene blue (MB) degradation by ·OH. A solution of MB (final concentration 100 μM) was mixed with different concentrations of chiral AuHNRs and chiral AuHNRs@CeO_2_, followed by the addition of Fenton reagent (1 mM Fe^2^⁺/10 mM H_2_O_2_). After reacting for 15 min, the absorbance at 665 nm was measured (A__sample_). Three controls were included: (1) Degradation control: MB + Fenton reagent (without nanomaterials), giving A__control_. (2) MB initial control: MB only (without Fenton reagent and nanomaterials), giving *A*__MB_. (3) Sample background control: Nanomaterials + MB (without Fenton reagent). The inhibition of MB degradation, which reflects ·OH scavenging capacity, was calculated as:Degradation inhibition (%) = [ (A__sample_ − A*_*_control_) / (A*_*_MB_ − A*_*_control_)] × 100%.

**·OH detection method**

·OH radical scavenging assay (Salicylic Acid Method): The hydroxyl radical scavenging activity was evaluated using a salicylic acid colorimetric method based on the Fenton reaction. Briefly, chiral AuHNRs and AuHNRs@CeO_2_ at varying concentrations were sequentially mixed with Fenton reagent (1 mM Fe^2^⁺/10 mM H_2_O_2_) in phosphate buffer (pH 7.4) and incubated in the dark at 37 °C for 10 min to generate and scavenge ·OH. Subsequently, salicylic acid solution (6 mM in ethanol) was added, and the mixture was further incubated at 37 °C in the dark for 30 min. The absorbance at 510 nm was measured (A__sample_). Three controls were included: (1) Positive control: Salicylic acid + Fenton reagent (without nanomaterials), giving A__Fenton_; (2) Sample background control: Nanomaterials + salicylic acid (without Fenton reagent), giving A__control_; (3) Reagent blank: Buffer only. After background correction, the scavenging rate was calculated as follows: Scavenging rate (%) = [ *(*A__Fenton_ − (A__sample_ − A__control_)) / *A__Fenton_* ] × 100%.

**Biocompatibility assessment of nanomaterials**

Hemolysis assay: A volume of 1.0 mL of rabbit blood was collected and then centrifuged at 3500 rpm for 5 min, and washed with PBS until the supernatant became colorless. The collected red blood cells were then co-incubated with different final concentrations (0, 25, 50, 100, and 200 μg/mL) of chiral AuHNRs and AuHNRs@CeO_2_ nanomaterials for 2 h. The experiment included a light exposure group (irradiation condition: 0.8 W/cm^2^ for 10 min) and a non-exposure group. Red blood cells treated with PBS (negative) and deionized water (positive) were used as controls. After incubation, the samples were centrifuged at 3500 rpm for 5 min, and the absorbance of the supernatant was measured at 545 nm using a microplate reader. The hemolysis rate was calculated according to the following formula:

$$\text{Hemolysis rate}\left( \text{\%} \right)\text{=}\frac{\text{OD}\text{sample}\text{ - OD}\text{negative}}{\text{OD}\text{positive}\text{- OD}\text{negative}}\text{×100\%}$$

**In vitro photothermal antibacterial activity of chiral AuHNRs and AuHNRs@CeO_2_**

The plate count method and live/dead staining experiment were used to evaluate the in vitro photothermal antibacterial activity of chiral AuHNRs and AuHNRs@CeO_2_ against *S. aureus*(CMCC (B)26003) and *E. coli*(ATCC25922). Briefly, bacterial suspensions (10^6^ CFU/mL) were mixed with chiral AuHNRs or AuHNRs@CeO_2_ at a final concentration of 100 µg/mL. Phosphate-buffered saline (PBS) was used as a negative control in place of the nanomaterials. The mixtures were then divided into two sets for each material: one set was kept in the dark (wrapped in foil and incubated in a shaker at 37 °C, 180 rpm), and the other set was irradiated with an 808 nm laser (0.8 W/cm^2^) for 10 min. After treatment, the mixtures were serially diluted, plated on agar, and incubated overnight. The number of colony-forming units (CFU) was counted. The antibacterial rate was calculated using the following formula: Antibacterial rate (%) = [ (CFU__control_ − CFU*_*_sample_) / CFU*_*_control_ ] × 100%

**Live/dead bacterial staining**

After distinct treatments, the bacterial suspension (10^7^ CFU/mL, 500 µL) was co‑stained in the dark for 15 min with equal volumes (500 µL each) of propidium iodide (PI, 0.03 mM) and N, N‑dimethylaniline N‑oxide (DMAO, 0.03 mM). After washing three times with PBS to remove unbound probes, the bacteria were observed using fluorescence microscope. All bacteria were effectively stained by DMAO (showing blue fluorescence), while only dead bacteria with compromised membrane integrity exhibited red fluorescence from PI.

**Morphological characterization of bacteria**

The morphologies of *E. coli* and *S.aureus* with various treatments were characterized by SEM. Briefly, the bacterial suspension (10^8^ CFU/ mL, 500 µL) was inoculated into each well with various treatments and incubated at 37 °C for 12 h under continuous shaking. Afterward, the bacteria were collected and fixed with glutaraldehyde (2.5% (*v/v*), 0.5 mL) at 4℃ for 20 min, and the fixed samples were sequentially dehydrated with gradient ethanol solutions (30-100%, *v/v*) for each 15 min. Finally, the SEM images of samples were captured after sputter-coating with gold.

**Crystal Violet and Live/Dead Staining for Biofilms**

Crystal violet staining for biofilm assessment was conducted as follows[5]: After incubating bacteria with different treatments for 24 h, the biofilms of *E. coli* and *S. aureus* were fixed with 70% methanol for 20 min. The fixed biofilms were stained with 0.1 mL of 1% (w/v) crystal violet solution for 5 min and then washed three times with PBS. After drying, the bound dye was dissolved in 0.5 mL of 33% (v/v) acetic acid, and the absorbance at 570 nm was measured using a microplate reader.

$$\text{Biomass}\left( \text{\%} \right)\text{=}\frac{\text{A}_{\text{Control}}\text{-}\text{A}_{\text{Sample}}}{\text{A}_{\text{Control}}}\text{×100\%}$$

Intracellular reactive oxygen species detection

Fluorescence microscopy: L929 cells were seeded into a 12-well plate and cultured for 24 h until fully adhered. The following control groups were established: Control group: complete medium only; H_2_O_2_ control group control: complete medium containing 2 mM H_2_O_2_; Experimental groups: 2 mM H_2_O_2_ + 100 µg/mL D-, L-, or DL-nanomaterials. All groups were treated simultaneously for 2 h. After discarding the supernatant, serum-free medium containing the fluorescent probe 2',7'-dichlorodihydrofluorescein diacetate (DCFH-DA, 10 µM) was added, followed by incubation at 37 °C in the dark for 30 min. The culture medium was then removed, and the cells were washed twice with pre-cooled PBS. Images were acquired using fluorescence microscopy.

Flow cytometry: L929 cells were seeded into a 6-well plate and cultured for 24 h until fully adhered. The following control groups were established: blank group (complete medium only), probe control group (serum-free medium only), H_2_O_2_ control group (complete medium containing 2 mM H_2_O_2_), and experimental groups (2 mM H_2_O_2_ + 100 µg/mL D-, L-, or DL-nanomaterials). All groups were treated simultaneously for 2 h. After discarding the supernatant, all wells except the blank group were treated with serum-free medium containing the fluorescent probe DCFH-DA (10 µM), followed by incubation at 37 °C in the dark for 30 min. The medium was then removed, and cells were washed twice with pre-cooled PBS, which were collected into 2 mL centrifuge tubes for analysis by flow cytometry.

**RT-qPCR Analysis**

RAW264.7 cells were seeded in a 6-well plate at a density of 2 × 10⁵ cells per well and cultured overnight at 37 °C in a 5% CO_2_ incubator. The cells were stimulated with 1 μg/mL LPS for 12 hours. Subsequently, chiral AuHNRs@CeO_2_ was added and incubated for an additional 12 hours.

L929 cells and HUVECs were seeded in a 6-well plate at a density of 1 × 10⁵ cells per well and cultured overnight at 37 °C in a 5% CO_2_ incubator. The cells were stimulated with H_2_O_2_ (L929 cells: 2 mM and HUVECs: 200 μM) for 12 hours. Subsequently, chiral AuHNRs@CeO_2_ was added and incubated for an additional 12 hours.

After this treatment, cells were washed with PBS, and total RNA was extracted using TRIzol reagent. The extracted RNA was treated with gDNA Eraser and reverse-transcribed into cDNA using the Evo M-MLV Reverse Transcription Premix kit, following the manufacturer's protocol. Quantitative PCR (qPCR) was then performed using the SYBR Green Pro Taq HS Premix qPCR kit on a real-time PCR system.

**Cellular phagocytosis assay**

L929 cells were seeded into 24-well culture plates and cultured for 24 h to allow cell attachment. The medium was then aspirated. Cells were subsequently treated for 2 h with complete medium containing 2 mM H_2_O_2_ (experimental groups) or with complete medium alone (control groups). After washing with PBS, all wells were incubated with medium containing 100 µg/mL FITC‑labeled chiral AuHNRs@CeO_2_ for 24 h. The medium was removed, cells were washed again, and then stained with 4’,6‑diamidino‑2‑phenylindole (DAPI). Finally, images were acquired using a high‑content imaging system (ImageXpress Micro Confocal).

**H&E staining**

Following euthanasia, tissue samples including the dorsal skin (wound site), heart, liver, spleen, lungs, and kidneys were collected and fixed in 4% paraformaldehyde. The tissues were then embedded in paraffin. Subsequently, the paraffin-embedded tissue blocks were sectioned into 4 μm thick slices. These sections were stained with hematoxylin and eosin (H&E) (Servicebio, China) to observe pathological changes in different tissues.

**Wound healing assay in vivo**

To evaluate the wound healing efficacy of chiral nanomaterials, this study established a skin wound infection model using female BALB/c mice. A full-thickness circular skin wound with a diameter of 8 mm was created on the dorsal side of each mouse. The wounds were inoculated with an *S. aureus* /*E. coli* suspension (10^7^ CFU/mL, 0.1 mL) to establish an infected full-thickness excisional wound model (designated as day 0). Mice with *S. aureus* /*E. coli* -infected wounds were randomly divided into four groups: control group (PBS), control + NIR group, chiral AuHNRs@CeO_2_ group, chiral AuHNRs@CeO_2_ + NIR group, levofloxacin hydrochloride (1 mg mL^-1^) group. Wounds in each group received corresponding treatments on days 1 and 3. Wound healing progress and mouse body weight were monitored on days 0, 3, 6, 9, and 12, and wound areas were quantified using ImageJ software.

**Biofilm elimination analysis**

After being treated with different groups, *S. aureus* or *E. coli* were incubated at 37 °C for 48 hours. The samples were then stained with the Live/Dead Bacterial Viability Kit for 15 minutes, and fluorescence images were obtained and examined using a Nikon super-resolution structured illumination microscopy (SIM) system.

**Assessment of Bacterial Membrane Potential**

Following the manufacturer's instructions for measuring membrane potential, the specific procedure was as follows: Escherichia coli and Staphylococcus aureus pretreated with the chiral nanomaterial under 808 nm NIR light irradiation or without light irradiation were adjusted to a bacterial density of 10⁶ cells/mL. DiOC2(3) was then added (at a final concentration of 30 μM) and incubated at room temperature for 30 minutes. Subsequently, detection was performed using a confocal laser scanning microscope.

**Detection of bacterial external structural integrity**

A bacterial suspension was incubated with chiral nanomaterials at 37 °C for 4 h, using PBS as a control.[6] After incubation, the mixture was centrifuged at 8000 rpm for 5 min. The absorbance of the supernatant was then measured at 260 nm and 280 nm to quantify the leakage of nucleic acids and proteins, respectively, which serves as an indicator of bacterial membrane integrity.

**In Vitro cytotoxicity assay**

L929 cells, mouse macrophage RAW264.7 cells, and human umbilical vein endothelial cells (HUVECs) were cultured in DMEM supplemented with 10% fetal bovine serum, 100 U/mL penicillin, and 100 μg/mL streptomycin at 37 °C in a 5% CO₂ atmosphere. Cells in the logarithmic growth phase were harvested and seeded into 96-well plates at an appropriate density and cultured for 24 h. Subsequently, chiral nanomaterials at various concentrations were added to the wells and incubated for another 24 h. After treatment, 20 μL of a 5 mg/mL 3-(4,5-dimethylthiazol-2-yl)-2,5-diphenyltetrazolium bromide (MTT) solution was added to each well. Following 4 h of incubation, the medium was carefully aspirated, and 150 μL of dimethyl sulfoxide (DMSO) was added to each well to dissolve the formazan crystals. The absorbance of each well was then measured at 570 nm using a microplate reader to assess cell viability.

**Wound healing assay**

L929 cells and HUVECs were seeded into a 12-well plate. When cell density reached approximately 80%, a scratch was made using a 200 μL pipette tip. The cells were then cultured in serum-free medium and treated with PBS or 100 μg/mL chiral nanomaterials for 12 h. Images were captured at 0 and 12 h to measure and record the width of the scratch wound.

**ITC assay**

ITC measurements were performed to evaluate the binding affinity between chiral AuHNRs@CeO_2_ (D-, L-, and DL-forms) and bacterial cells. D-, L-, and DL-AuHNRs@CeO_2_ solutions (0.06 mg/mL, 50 μL) were loaded into the syringe, and *E. coli* or *S. aureus* suspensions (0.01 mg/mL, 300 μL) were placed in the sample cell. Each experiment consisted of 20 sequential injections (2 μL each) at 25 °C, 350 rpm, with a 120 s interval between injections. Control experiments were performed by titrating the corresponding AuHNRs@CeO₂ solutions into blank PBS buffer to subtract the heat of dilution. Data were fitted using a one-site binding model to obtain the binding constant (Ka), enthalpy change (ΔH), and entropy change (ΔS).

**Statistical Analysis**

Statistical analyses were conducted using GraphPad Prism 10, and results were expressed as mean ± SD. Multi-group comparisons were performed using one-way ANOVA followed by Dunnett's post-hoc test. For wound healing and bacterial colony counting in animal experiments, two-way repeated measures ANOVA was used, followed by Tukey's post-hoc test for multiple comparisons. Statistical significance was defined as *p < 0.05, **p < 0.01, ***p < 0.001, (n = 3)

Supporting Figures and Tables

**Fig. S1** UV-Vis spectrum of AuNRs**.**

**Fig. S2** Structural formula of (a) L-Cys and (b) D-Cys.

**Fig. S3** SEM images. (a) D-AuHNRs; (b) L-AuHNRs.

**Fig. S4** Bactericidal effect of chiral AuHNRs and chiral AuHNRs loaded with different concentrations of CeO_2_ against *S. aureus.*

**Fig. S5** The g-factor spectra of DL-/L-/D-AuHNRs and DL-/L-/D-AuHNRs@CeO_2_.

**Fig. S6** The g-factor spectra of chiral AuHNRs before and after mPEG-SH modification.

**Fig. S7** Length distribution of L‑AuHNRs@CeO_2_ (a), D‑AuHNRs (c) and L‑AuHNRs (e). Diameter distribution of L‑AuHNRs@CeO_2_ (b), D‑AuHNRs and (d) L‑AuHNRs(f) (n = 100).

**Fig. S8.** FTIR spectra of mPEG-SH, L -AuHNRs@CeO_2_, and L -AuHNRs @CeO_2_ (mPEG-SH) (a); FTIR spectra of mPEG-SH, DL -AuHNRs@CeO_2_, and DL -AuHNRs @CeO_2_ (mPEG-SH) (b).

**Fig. S9.** Zeta potential of DL-/L-/D-AuHNRs@CeO_2_ before and after modification.

**Fig. S10** The XPS spectra of DL/L/D-AuHNRs@CeO_2_.

**Fig. S11** HRTEM image and mapping of L-AuHNRs@CeO_2_ (a) and DL-AuHNRs@CeO_2_ (b).

**Fig. S12** UV‑Vis spectra of DL-/L-/D-AuHNRs@mPEG-SH at different time points.

**Fig. S13** Stability test. CD (a) and g-factor (b) changes of DL-/L-/D-AuHNRs@mPEG-SH at different time points.

**Fig. S14** Heating curves under 808 nm laser irradiation, cooling curves after laser shutdown, and plots of -lnθ versus time derived from cooling time profiles (0.8 W/cm^2^, 10 min). D-AuHNRs (a), L-AuHNRs (b), DL-AuHNRs (c) and DL-AuHNRs@CeO_2_ (d).

**Fig. S15** Temperature rise curves of nanomaterials at different concentrations under 808 nm laser irradiation (0.8 W/cm^2^, 10 min) DL-AuHNRs and DL-AuHNRs@CeO_2_.

**Fig. S16** Temperature rise curves of different nanomaterials under 808 nm laser irradiation at various laser power densities (0.8 W/cm^2^, 10 min). D-AuHNRs (a), L-AuHNRs (b), DL-AuHNRs (c) and DL-AuHNRs@CeO_2_ (d).

**Fig. S17** Thermal stability measurement during five consecutive laser on-off cycles (0.8 W/cm^2^, 10 min). (a) D-AuHNRs, (b) L-AuHNRs, (c) DL-AuHNRs, (d) DL-AuHNRs@CeO_2_.

**Fig. S1****8** DPPH radical scavenging rates of D-AuHNRs (a), L-AuHNRs (b), DL-AuHNRs (c), L-AuHNRs@CeO_2_ (d) and DL-AuHNRs@CeO_2_ (e) at different gradient concentrations.

**Fig. S19** ABTS·^+^ radical scavenging rates of D-AuHNRs (a), L-AuHNRs (b), DL-AuHNRs (c), L-AuHNRs@CeO_2_ (d) and DL-AuHNRs@CeO_2_ (e) at different gradient concentrations.

**Fig. S20** ·OH scavenging rates of D-AuHNRs (a), L-AuHNRs (b), DL-AuHNRs (c), L-AuHNRs@CeO_2_ (d) and DL-AuHNRs@CeO_2_ (e) nanocomposites at gradient concentrationsdetected by methylene blue (MB) method.

**Fig. S21** Hydroxyl radical scavenging rates of D-AuHNRs (a), L-AuHNRs(b), DL-AuHNRs (c), L-AuHNRs@CeO_2_ (d) and DL-AuHNRs@CeO_2_ (e) nanocomposites at gradient concentrations by 3,3',5,5'-tetramethylbenzidine (TMB) method.

**Fig. S22** Hydroxyl radical scavenging rates of D-AuHNRs (a), L-AuHNRs (b), DL-AuHNRs (c), D-AuHNRs@CeO_2_ (d), L-AuHNRs@CeO_2_ (e) and DL-AuHNRs@CeO_2_ (f) nanocomposites at gradient concentrations by salicylic acid colorimetric method.

**Fig. S23** Hemolysis rate of unmodified chiral nanomaterials after co-incubation with red blood cells.

**Fig. S24** Hemolysis rate of PEG-modified chiral nanomaterials at different concentrations before and after light irradiation following co-incubation with RBCs. (a) D-AuHNRs, (b) L-AuHNRs, (c) DL-AuHNRs, (d) D-AuHNRs@CeO_2_, (e) L-AuHNRs@CeO_2_, and (f) DL-AuHNRs@CeO_2_.

**Fig. S25** Antibacterial effects of chiral AuHNRs. (a) Photographs of agar plates inoculated with *S. aureus* and *E. coli* after different treatments, (b) Bacterial viability of *S. aureus* following various therapeutic methods, (c) Bacterial viability of *E. coli* following various therapeutic methods (****p* < 0.001, ***p* < 0.01, **p* < 0.05).

**Fig. S26** Live/dead bacterial staining of *S. aureus* and *E. coli* after different treatments (green: live bacteria, red: dead bacteria; scale bar: 50 μm).

**Fig. S27** Inhibitory effects of chiral AuHNRs on bacterial biofilms**.** (a) Crystal violet staining assay for *S. aureus* and *E. coli* following various therapeutic methods, (b) Biofilm viability of *S. aureus* after different therapeutic methods, (c) Biofilm viability of *E. coli* after different therapeutic methods (***p < 0.001, ***p* < 0.01, *p *<* 0.05).

**Fig. S28** Disruption effects of chiral AuHNRs on bacterial biofilms. (a) Crystal violet staining assay for *S. aureus* and *E. coli* following various therapeutic methods, (b) Biofilm viability of *S. aureus* after different therapeutic methods, (c) Biofilm viability of *E. coli* after different therapeutic methods (****p* < 0.001, ***p* < 0.01, **p <* 0.05).

**Fig. S29** 3D confocal microscopy reconstruction images showing structural changes in bacterial biofilms after different treatments (scale bar: 200 μm)

**Fig. S30** SEM images of *S. aureus* and *E. coli* after treatment with different groups (scale bar: 1 μm).

**Fig. S31** Temperature changes of *S. aureus* solutions during treatment with control (PBS), chiral AuHNRs and chiral AuHNRs@CeO_2_ at a concentration of 100 µg/mL under 808 nm laser irradiation.

**Fig. S32** ITC titration curves and S-shaped binding isotherms of (a) D-AuHNRs@CeO_2_, (b) L-AuHNRs@CeO_2_, and (c) DL-AuHNRs@CeO_2_ with E. coli at 25 °C. The top panels show the raw thermograms (heat rate vs. time), and the bottom panels show the integrated binding isotherms (normalized heat vs. molar ratio). The solid lines represent the best fit using a one-site binding model.

**Fig. S33** Cell viability of different cell types treated with chiral AuHNRs@CeO_2_. Cell viability of L929 cells, RAW264.7 cells and HUVECs after treatment with D-AuHNRs@CeO_2_ (a), L-AuHNRs@CeO_2_ (b) and DL-AuHNRs@CeO_2_ (c).

**Fig. S34** Optimization of oxidative stress conditions: Cell viability of L929 cells stimulated with different concentrations of H_2_O_2_ for various durations. (a) 1 h, (b) 2 h, and (c) 4 h.

**Fig. S35** Interaction images of L929 cells with chiral AuHNRs@CeO_2_ labeled with FITC (scale bar: 100 μm).

**Fig. S36** *In vivo* antibacterial activity of chiral AuHNRs@CeO₂ against ***E. coli* infection** in BALB/c mice**.** (**a)** Schematic representation of the wound healing process over time for each treatment group. (**b)** Wound simulation diagrams for each group. (c) Bacterial colony images collected from wound sites of mice in each treatment group.

**Fig. S37** *In vivo* antibacterial activity of chiral AuHNRs@CeO_2_ against ***E. coli* infection** in BALB/c mice**.** (**a)** Quantitative assessment of wound closure rates across treatment groups. (**b)** Quantification of bacterial colony counts from wound sites (n = 5) (***p < 0.001, **p < 0.01, *p < 0.05).

**Fig. S38** Body weight changes of mice treated with different chiral AuHNRs@CeO_2_ during skin wound healing.

**Fig. S39** Body weight changes of mice treated with different chiral AuHNRs@CeO_2_ during skin wound healing.

**Fig. S40** Histological analysis of major organs in mice by H&E staining (scale bar: 300 μm).

**Table S1.** **Basic Morphological Parameters**

| **Samples** | **Length (nm)** | | **Diameter (nm)** | **Aspect Ratio (AR)** |
| --- | --- | --- | --- | --- |
| D-AuHNRs | | 92.5 0± 0.80 | 23.1 ± 0.20 | 4.00 ± 0.55 |
| L-AuHNRs | | 92.5 ± 1.02 | 23.3 ± 0.30 | 3.97 ± 0.07 |
| D-AuHNRs@CeO_2_ | | 98.9 ± 1.11 | 25.1 ± 0.22 | 3.94 ± 0.06 |
| L-AuHNRs@CeO_2_ | | 97.5 ± 1.82 | 25.2 ± 0.16 | 3.87 ± 0.88 |

**Table S2. Gene primer sequences used in RT-qPCR**

| **Gene** | **Forward primers** | **Reverse primers** |
| --- | --- | --- |
| iNOS | 5’-TTT GCT TCC ATG CTA ATG CGA AAG-3’ | 5’-GCT CTG TTG AGG TCT AAA GGC TCC G-3’ |
| IL-6 | 5’-TGA AAA GAT GCT GGT GAA AGT G-3’ | 5’-TCT GAA GAG GGA GTT GAG GTA GT-3’ |
| CD206 | 5’-CAT GAG GCT TCT CTT GCT TCT G-3’ | 5’-TTG CCG TCT GAA CTG AGA TGG -3’ |
| Arg-1 | 5’-CTC CAA GCC AAA GTC CTT AGA G-3’ | 5’-AGG AGC TGT CAT TAG GGA CAT C-3’ |
| GAPDH | 5’-ATA CGG CTA CAG CAA CAG GG-3’ | 5’-GCC TCT CTT GCT CAG TGT CC-3’ |
| CAT | 5’-AGC GAC CAG ATG AAG CAG TG-3’ | 5’-TCC GCT CTC TGT CAA AGT GTG-3’ |
| TGF-β1 | 5’-CTC CCG TGG CTT CTA GTG C-3’ | 5’-GCC TTA GTT TGG ACA GGA TCT G-3’ |
| MMP1^*^ | 5’-TCG CCC ATC ATC AAG TTC CC-3’ | 5’-GGG CAG CCA TAG AAG GTG TT-3’ |
| GAPDH^*^ | 5’-CGC TCT CTG CTC CTC CTG TT-3’ | 5’-CCA TGG TGT CTG AGC GAT GT-3’ |

* Human gene

**References**

[1] L.T. Li, Y.S. Feng, C. Liu, Y.Y. Xiong, H.F. Shi, Q.Y. Liu, Q.S. Li, X.H. Zhao, J. Wang, Single-Stranded Nucleic Acid-Induced Aggregation of Intrinsically Chiral Helical Gold Nanorods for Biosensing Applications, Anal. Chem. 97 (2025) 17624-17630.

[2] L.T. Li, S. Wen, Q.Y. Liu, H.F. Shi, M. Huang, C. Liu, L. Zhan, X.H. Zhao, H.Y. Zou, C.Z. Huang, Intrinsic Chirality Modulation and Biosensing Application of Helical Gold Nanorods by Anisotropic Etching, Anal. Chem. 97 (2025) 319-327.

[3] F. Wang, W. Yang, Q. Ding, X. Xing, L. Xu, H. Lin, C. Xu, S. Li, Chiral Au@CeO_2_ Helical Nanorods with Spatially Separated Structures for Polarization-Dependent N_2_ Photofixation, Angew. Chem., Int. Ed. 64 (2025) e202415031.

[4] X. Wang, X. Qin, Y. Liu, Y. Fang, H. Meng, M. Shen, L. Liu, W. Huan, J. Tian, Y.W. Yang, Plasmonic Supramolecular Nanozyme‐Based Bio‐Cockleburs for Synergistic Therapy of Infected Diabetic Wounds, Adv. Mater. 36 (2024) 2411194.

[5] Y. Wang, K. Liu, W. Wei, H. Dai, A Multifunctional Hydrogel with Photothermal Antibacterial and Antioxidant Activity for Smart Monitoring and Promotion of Diabetic Wound Healing, Adv. Funct. Mater. 34 (2024) 2402531.

[6] X. Huang, C. Lu, W. Zhang, L. Liu, Z. Zha, Z. Miao, Chiral Sulfur Nanosheets for Dual-Selective Inhibition of Gram-Positive Bacteria, ACS Nano 17 (2023) 14893-14903.
